# The liver-brain axis, from its function to preventive therapeutic strategies in diseases

**DOI:** 10.3389/fendo.2026.1858091

**Published:** 2026-06-26

**Authors:** Yaoyao Chen, Shangcheng Xu, Tao Sun

**Affiliations:** 1 Center for Precision Medicine, School of Medicine and School of Biomedical Sciences, Huaqiao University, Xiamen, China; 2 GeneYoung Biopharmaceuticals, Shenzhen, China

**Keywords:** central nervous system, diabetes, liver-brain axis, obesity, psychiatric disorders

## Abstract

The liver-brain axis (LBA) represents a bidirectional communication network between the liver and the central nervous system (CNS), governed by integrated neural, humoral, and immune signaling pathways. Emerging evidence indicates that the liver functions not merely as a passive metabolic organ subordinate to central commands, but rather as a dynamic hub that actively senses and modulates peripheral neuroimmune responses. We here first delineate the fundamental communication mechanisms and the transport kinetics of signaling molecules governing LBA interactions. We then examine the profound species-specific disparities between humans and mice—particularly regarding signaling mediators, blood-brain barrier (BBB) architecture, and the hepatic immune microenvironment. From a pathophysiological perspective, we establish that LBA dysfunction serves as a core driver of obesity, diabetes, and their multisystemic sequelae, including cardiovascular diseases and psychiatric disorders such as anxiety and depression. Finally, we highlight recent therapeutic advances targeting the LBA for the management of metabolic dysfunction-associated steatotic liver disease (MASLD), metabolic dysfunction-associated steatohepatitis (MASH), atherosclerosis, and associated psychiatric conditions, thereby underscoring the immense clinical potential of LBA-targeted interventions.

## 1 Introduction

**Figure 1:**
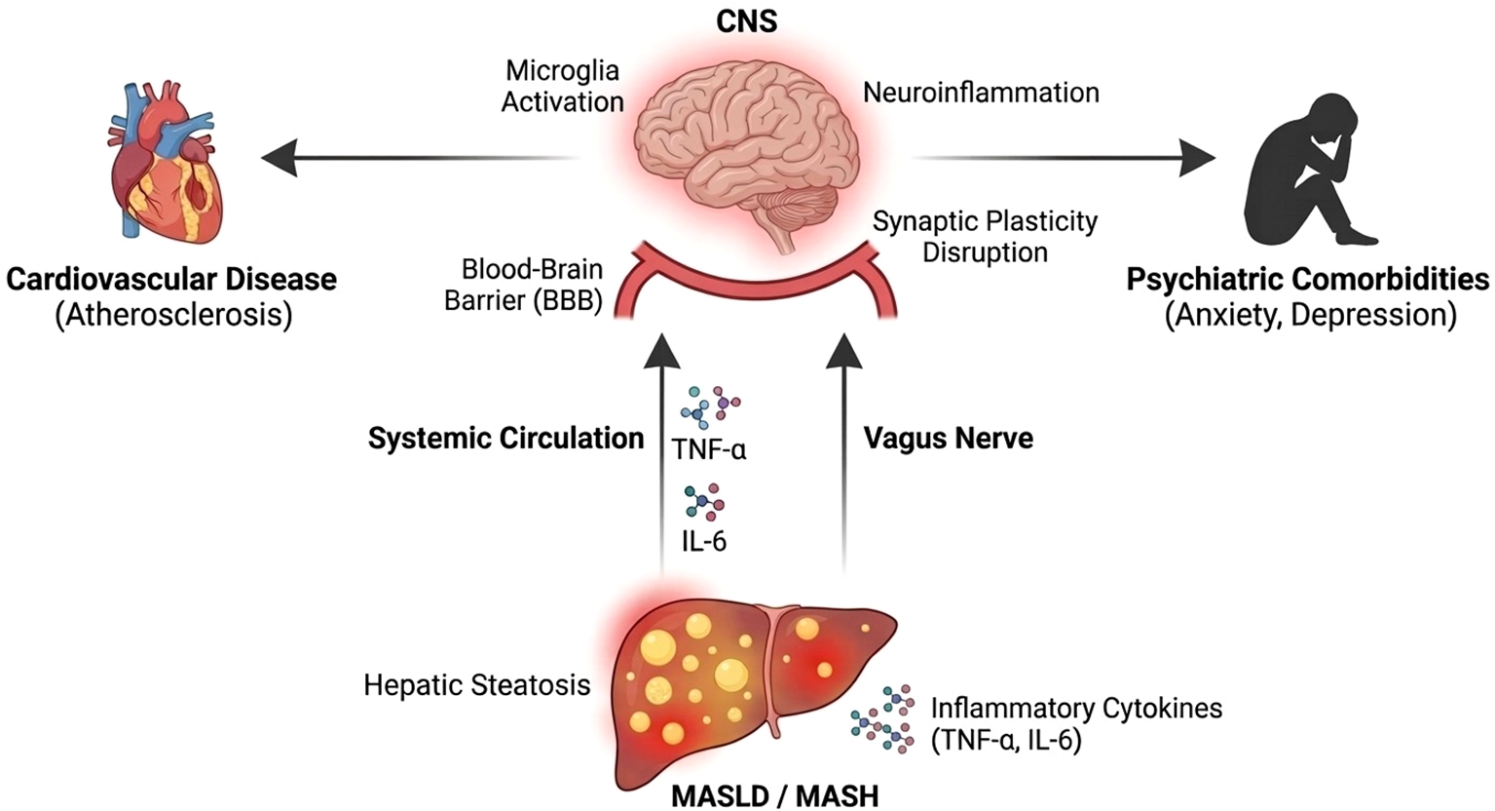
Mechanistic overview of the Liver-Brain Axis (LBA) dysregulation in driving cardiometabolic and psychiatric complications. In the context of metabolic dysfunction-associated steatotic liver disease (MASLD) and steatohepatitis (MASH), the steatotic liver serves as a primary driver of systemic inflammation through the continuous secretion of pro-inflammatory cytokines, particularly TNF-α and IL-6. These peripheral inflammatory signals are transmitted to the central nervous system (CNS) via two principal pathways: humoral transport across the blood-brain barrier (BBB) and neural transmission via afferent vagal fibers. Within the brain, these mediators trigger robust neuroinflammation, characterized by profound microglial activation and impaired synaptic plasticity. Ultimately, this centrally driven dysregulation precipitates diverse pathological sequelae, exacerbating cardiovascular conditions, such as atherosclerosis, and fueling the development of psychiatric comorbidities.

Obesity and diabetes have emerged as intertwined global pandemics. The global population of overweight and obese individuals surpassed 2.6 billion, encompassing 2.11 billion adults and 493 million children and adolescents (1). In 2021, the estimated global prevalence of diabetes among adults aged 20–79 reached 10.5% (2). Beyond precipitating peripheral complications—such as cardiovascular diseases and metabolic diseases and non-alcoholic fatty liver disease— obesity and diabetes are now recognized as established risk factors for neurocognitive impairment and mood disorders. For example, a large-scale analysis of nearly 424, 000 UK Biobank participants revealed profound associations between obesity, adverse neuropsychiatric outcomes, and structural brain alterations (3). Similarly, large-scale longitudinal cohorts demonstrate that individuals with diabetes face a markedly elevated risk of accelerated cognitive decline and incident major depressive disorder, driven mechanistically by chronic neurovascular dysfunction and cerebral insulin resistance (4, 5) (**Figure 1**).

The liver, functioning as the body’s principal metabolic hub, orchestrates nutrient metabolism, detoxification, and energy homeostasis (6). Concurrently, the brain serves as the master integrator of neural and endocrine signals, regulating cognition, emotion, and systemic physiological balance through intricate neural networks (7). Historically, these two organs have been studied in isolation, largely regarded as discrete functional entities. Recent advances in systems biology and emerging clinical data fundamentally challenge this compartmentalized paradigm. It is now accepted that the liver and brain form a dynamic, bidirectional communication network—termed the liver-brain axis (LBA)—that continuously coordinates systemic metabolic and neuroimmune responses (8). Elucidating the role of LBA not only deepens our mechanistic understanding of inter-organ crosstalk but also provides novel therapeutic insights into the pathophysiology of intertwined metabolic and neuropsychiatric disorders.

It appears that the LBA orchestrates critical inter-organ crosstalk via a dynamic bidirectional signaling network integrating neural, metabolic, and immune pathways (9–11). The pathophysiological relevance of this axis is classically exemplified by hepatic encephalopathy (HE). When hepatic function is severely compromised, systemic ammonia evades hepatic clearance and penetrates the blood-brain barrier (BBB). Subsequent hyperammonemia precipitates a cascade of neurotoxic events—specifically astrocyte swelling, oxidative stress, and neurotransmitter dysregulation—ultimately manifesting as profound neurocognitive and psychiatric impairments (12–14).

In summary, this review elucidates the fundamental role of the LBA in maintaining systemic homeostasis and evaluates emerging therapeutic strategies targeting this axis for associated neurometabolic disorders.

## 2 Communications between the liver and the central nervous system

**Figure 2:**
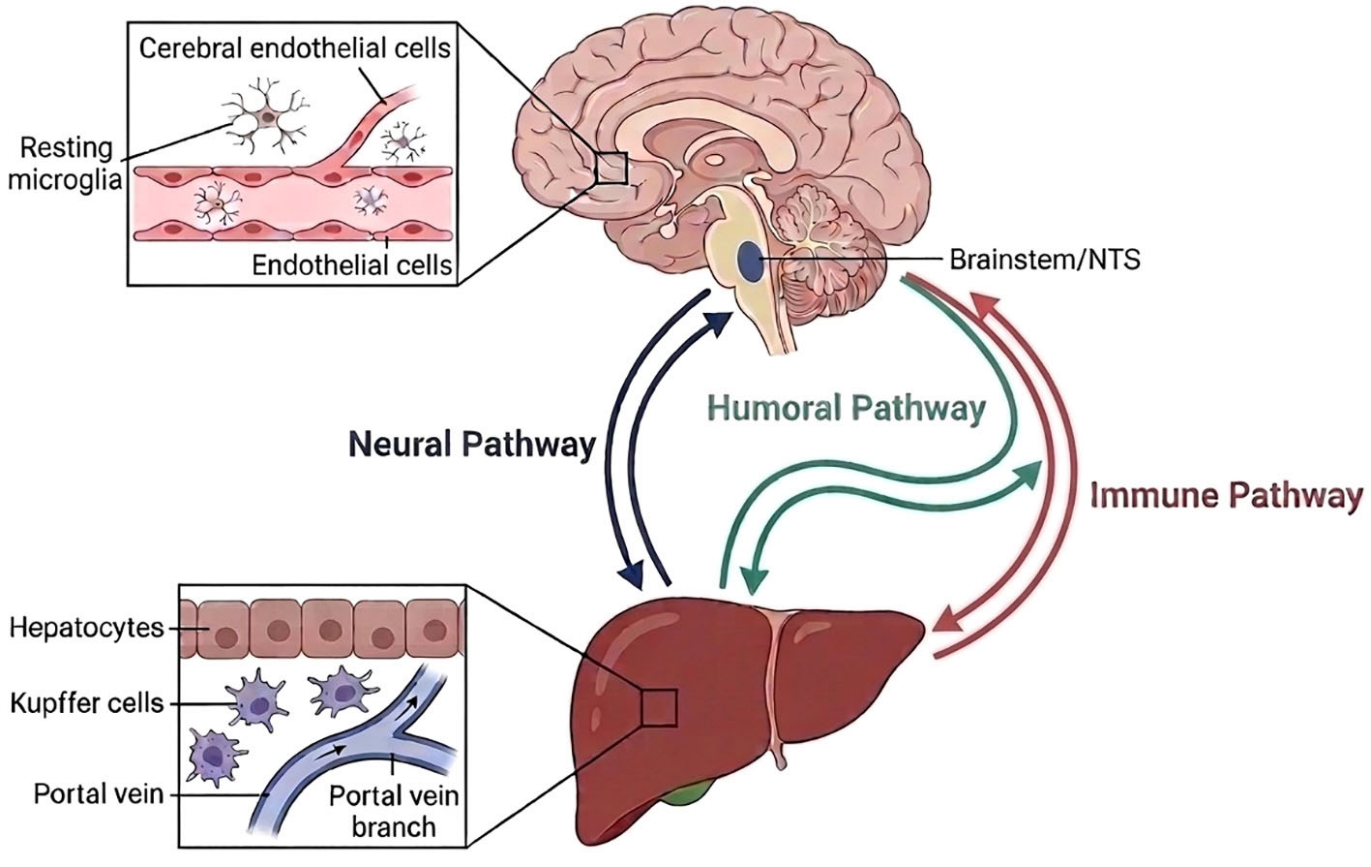
Three communication pathways between the liver and the central nervous system. The physiological and pathological crosstalk between the liver and the CNS is orchestrated by a highly integrated, bidirectional tripartite network encompassing neural, humoral, and immune axes. Hepatic afferents transmit critical visceral signals via the vagus nerve directly to the brainstem’s nucleus of the solitary tract (NTS). Complementing this hardwired connection, the hepatic microenvironment—comprising parenchymal hepatocytes and resident Kupffer cells—continuously monitors metabolic fluctuations. Upon activation, these cells secrete circulating mediators that propagate peripheral signals to the cerebral milieu, where they engage vascular endothelial cells and resident microglia. Ultimately, this multifaceted communication paradigm redefines the liver: rather than serving merely as a passive metabolic effector, it functions as a dynamic neuroimmune hub capable of actively dictating CNS responses and maintaining systemic homeostasis.

The LBA functions as a dynamic, bidirectional communication network that integrates neural, humoral, and immune pathways. Within this architecture, ascending hepatic afferent signals continuously relay peripheral metabolic status to the central nervous system (CNS), facilitating essential neurovisceral integration and the maintenance of systemic homeostasis (15) (**Figure 1**). Reciprocally, the brain exerts top-down efferent control over the liver via sympathetic and parasympathetic (vagal) pathways, finely tuning hepatic metabolism and secretory functions (16, 17) (**Figure 2**).

Anatomically, the somata of hepatic vagal afferent neurons predominantly reside within the left nodose ganglion. Their axons extend peripherally to innervate portal vein branches within the hepatic parenchyma, particularly enriching the periportal and midlobular regions. This strategic localization facilitates the precise chemosensory monitoring of portal venous blood composition (18–20). Upon activation, these peripheral metabolic signals are relayed centrally to the brainstem via the nucleus of the solitary tract (NTS), which subsequently routes this viscerosensory information to higher-order autonomic and limbic structures, notably the hypothalamus and amygdala (21).

Second, the humoral axis facilitates the systemic distribution of liver-derived metabolites, underscoring the liver’s role as an endocrine organ in inter-organ communication (22). Upon reaching the CNS, these circulating factors modulate critical neurological processes, such as synaptic plasticity (23). Mechanistically, these molecules exert their effects either by traversing the blood-brain barrier (BBB) via specific transporters (24) or by initiating peripheral receptor-mediated signaling cascades (25, 26) (**Figure 2**).

Third, the immune pathway underscores the liver’s capacity as a primary immunological hub. During hepatic injury or pathological stress, resident hepatic macrophages (Kupffer cells) secrete a potent repertoire of inflammatory cytokines—most notably tumor necrosis factor-alpha (TNF-α) and interleukin-6 (IL-6)—into the systemic circulation. These circulating mediators effectively relay the peripheral immune status to the CNS by activating cerebral vascular endothelial cells and propagating neuroinflammation. Consequently, this cascade fundamentally drives adverse neurobehavioral and neurodegenerative outcomes (27, 28) (**Figure 2**).

## 3 Signaling molecules between the liver and the central nervous system

Hepatokines constitute a specialized class of liver-derived endocrine proteins (**Table 1**). Synthesized by hepatocytes and secreted into the systemic circulation, they orchestrate inter-organ metabolic crosstalk, exerting regulatory effects across the liver, adipose tissue, and the CNS (29). Within this endocrine network, fibroblast growth factor 21 (FGF21) and insulin-like growth factor 1 (IGF-1) warrant particular emphasis due to their critical roles as circulating mediators in the liver-brain axis.

**Table 1:** Liver–brain axis signaling molecules: pathophysiological mechanisms and translational implications.

Signal category	Signaling molecule	Hepatic/peripheral source	BBB transport mechanism	CNS target receptors & primary brain regions	Downstream signaling pathways & neurobiological effects	Associated pathologies	Potential therapeutic strategies	Reference
Hepatokines	FGF21	Hepatocytes (nutritional/metabolic status)	Endocytosis/Active transport	β-Klotho + FGFR (VMH, etc.)	Suppresses simple sugar appetite; increases protein consumption	Obesity, metabolic syndrome, eating disorders	FGF21 analogs (e.g., Pegbelfermin)	(31–37)
	IGF-1	Hepatocytes (growth hormone)	Receptor-mediated transcytosis	IGF-1R (Neurons, astrocytes)	PI3K/Akt/GSK-3β pathway activation; Aβ clearance; tau hyperphosphorylation suppression	Cerebral ischemia, Alzheimer’s disease, cognitive decline	Intranasal/carrier-coupled IGF-1 delivery.	(38–47)
	LCN2	Hepatocytes (elevated in NASH)	Crosses BBB	LCN2R (Microglia)	Microglial activation; neuroinflammation	NASH-associated cognitive impairment	Targeted LCN2 blockade	(55, 56)
Pro-inflammatory Mediators	TNF-α, IL-1β, IL-6	Kupffer cells, hepatic stellate cells, hepatocytes	CVO diffusion, BBB transporters, monocyte infiltration	TNFR1/2, IL-1R1, IL-6R (Astrocytes, microglia, neurons)	NF-κB & JAK/STAT activation; neuroinflammation; synaptic disruption	NASH cognitive impairment, cirrhosis	TNF-α inhibitors (e.g., Etanercept)	(48–54)
Bile Acids	Unconjugated & Conjugated Bile Acids (DCA, LCA, etc.)	Liver synthesis, microbiota metabolism	Passive diffusion (unconjugated); OATPs transport (conjugated)	TGR5, FXR (Neurons, glia)	cAMP signaling & FXR modulation; microglial polarization	Hepatic encephalopathy (HE), primary biliary cholangitis	TGR5 agonists (e.g., INT-777), FXR ligands	(57–63)
Neurotoxins	Ammonia (NH_3_/NH_4_^+^)	Intestinal microbiota; impaired hepatic clearance	Passive diffusion (NH_3_); K^+^ channels/NKCC1 (NH_4_^+^)	Astrocytes (Glutamine synthetase)	Osmotic swelling, mitochondrial dysfunction, ROS, neurotransmitter dysregulation	HE, acute liver failure	Lactulose, rifaximin, L-ornithine-L-aspartate	(64–67)
Xenobiotics	Drugs/Xenobiotics	Intestinal absorption; impaired CYP450 clearance	Passive diffusion, BBB hyperpermeability	Neurons, astrocytes	Synergistic toxicity with ammonia; neurotransmitter disruption	Cirrhosis-related pharmacological encephalopathy	Individualized dosing based on CYP450; BBB efflux stimulators	(68–74)
Neural Pathway Mediators	IL-1β, TNF-α (Local paracrine)	Local hepatic immune cells	Paracrine diffusion (No BBB crossing required)	IL-1R1, TNFR (Hepatic vagal afferents)	Vagal afferent depolarization; NTS-to-amygdala signal propagation	Anxiety, depression, sickness behavior	Vagus nerve stimulation (VNS), peripheral immune blockade	(9, 75–79)

**Figure 3:**
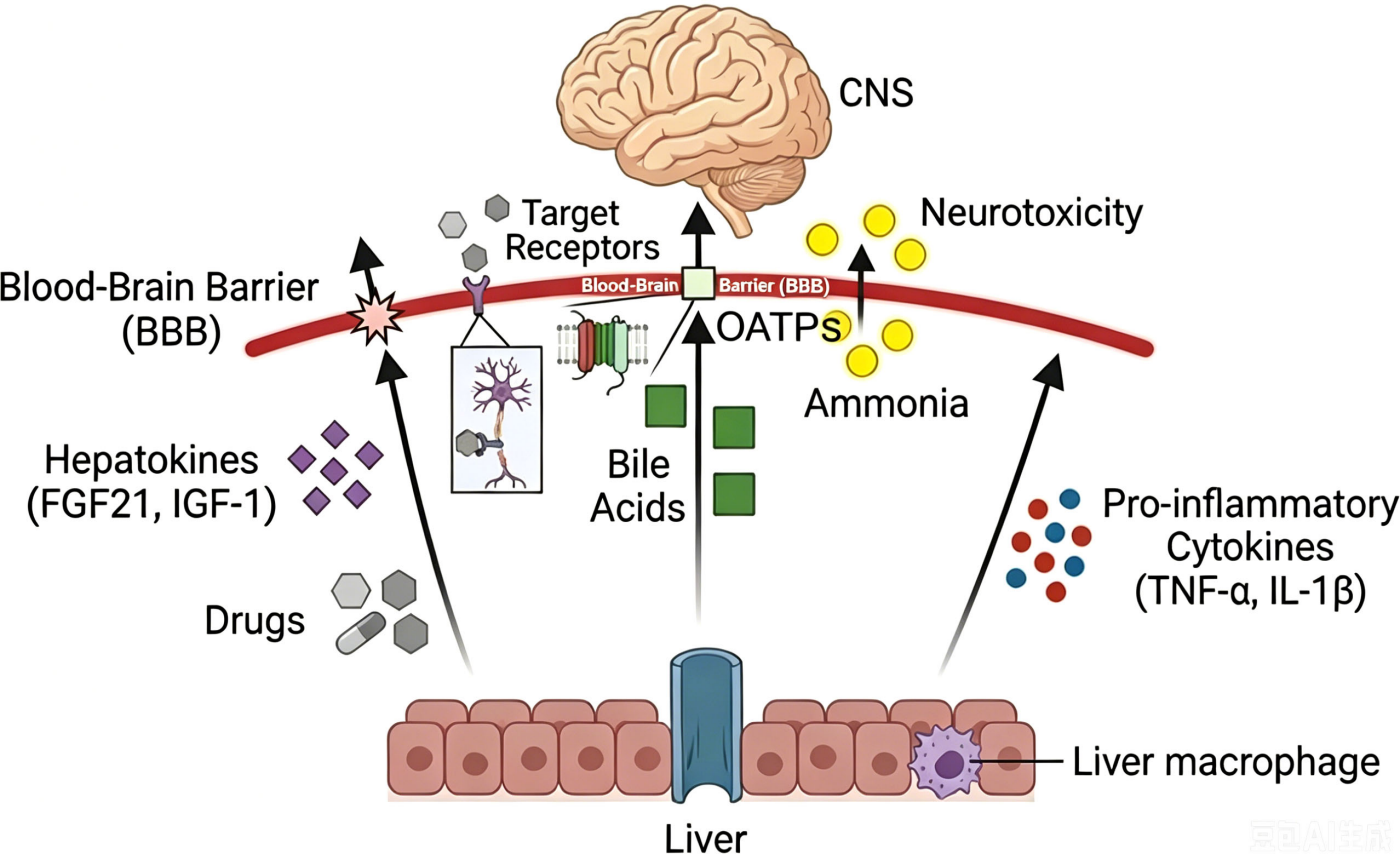
The mechanism of substance transportation between the liver and the brain. This schematic illustrates the diverse humoral, metabolic, and immune signaling pathways through which the liver modulates the CNS function. Upon entering the systemic circulation, liver-derived molecules interact with receptors at, or traverse, the BBB to exert central effects (1). Immune signaling: Hepatic macrophages (Kupffer cells) secrete pro-inflammatory cytokines, specifically TNF-α and IL-1β, which act as critical humoral mediators propagating peripheral inflammatory signals to the brain. (2) Metabolic and pharmacological signaling: Hepatokines (e.g., FGF21 and IGF-1) and exogenously administered drugs can cross the BBB or bind directly to specific receptors on central neurons. Additionally, circulating bile acids are transported across the BBB via specific solute carriers (e.g., OATPs). (3) Pathological neurotoxicity: During liver dysfunction, impaired hepatic clearance leads to systemic ammonia accumulation. This excess ammonia subsequently permeates the BBB, directly inducing severe neurotoxicity—a pathophysiological hallmark of hepatic encephalopathy. Collectively, these pathways underscore the active role of the hepatic microenvironment in shaping central homeostatic and neuroinflammatory landscapes.

Functioning as a primary liver-to-brain endocrine messenger, FGF21 relays systemic nutritional and metabolic status to the CNS. It crosses the BBB via active transport mechanisms. Its central signaling is strictly dependent upon the obligate co-receptor β-Klotho in complex with fibroblast growth factor receptors (FGFRs). This receptor complex is prominently expressed across key neuroanatomical hubs—including the suprachiasmatic nucleus, lateral hypothalamus, amygdala, ventral tegmental area, and numerous midbrain and brainstem nuclei (30, 31). By engaging these diverse neural circuits, FGF21 robustly modulates macronutrient preference. Specifically, it suppresses simple sugar appetite via direct action on glutamatergic neurons within the ventromedial hypothalamus (VMH) (32, 33), while concurrently driving a compensatory increase in protein consumption (34, 35). Because these pathways implicate FGF21 in the pathogenesis of obesity, metabolic syndrome, and eating disorders, they have spurred the development of FGF21 analogs (e.g., pegbelfermin) aimed at correcting metabolic dysregulation and aberrant dietary preferences (**Figure 3**).

Similarly, IGF-1—a liver-derived polypeptide regulated by growth hormone—crosses the BBB via receptor-mediated transcytosis (**Table 1**). Upon entering the CNS, it binds to the IGF-1 receptor (IGF-1R), which is broadly expressed on neurons, astrocytes, and glia, to mediate diverse neurobiological functions. The primary downstream consequence of this binding is the activation of the PI3K/Akt cascade, a central regulator of cellular survival, proliferation, and metabolism (36–39). For instance, following cerebral ischemia-reperfusion injury, IGF-1 exerts profound neuroprotective effects by activating the PI3K/Akt pathway, which subsequently modulates the Hippo/YAP signaling cascade via molecular crosstalk (36). Beyond acute injury, clinical evidence indicates that cerebral IGF-1 levels correlate strongly with cognitive function (40). This relationship is particularly pivotal in the context of Alzheimer’s disease (AD), a neurodegenerative pathology characterized by amyloid-beta (Aβ) deposition and aberrant tau phosphorylation (41). Under physiological conditions, circulating IGF-1 facilitates Aβ clearance from the CNS. Furthermore, by activating the Akt/GSK-3β pathway, IGF-1 suppresses the generation of hyperphosphorylated tau and the subsequent formation of neurofibrillary tangles, thereby preserving neuronal integrity and synaptic function (42, 43), Given these potent neuroprotective properties, advanced therapeutic strategies—such as intranasal administration and carrier-coupled delivery systems—are actively being investigated to elevate brain IGF-1 levels and attenuate AD progression (44) (**Figure 3**).

Under conditions of hepatic injury or disease, the liver serves as a critical amplifier of systemic inflammatory responses (**Table 1**). Specifically, during acute liver failure, chronic liver disease, or cirrhosis, activated Kupffer cells and hepatic stellate cells recognize damage-associated molecular patterns (DAMPs) and secrete pro-inflammatory cytokines, such as TNF-α and IL-1β (**Table 1**). These cytokines subsequently bind to hepatocyte surface receptors, triggering intracellular signaling cascades, notably the NF-κB and JAK/STAT pathways. Consequently, hepatocytes synthesize acute-phase proteins, hepatokines, and secondary cytokines, including IL-6. These mediators propagate inflammatory signals from the liver to the brain primarily via humoral pathways, which include passive diffusion at circumventricular organs (CVOs) and carrier-mediated transport across the BBB. Upon reaching the CNS, these cytokines act on their cognate receptors—TNFR1/2, IL-1R1, and IL-6R—expressed on astrocytes, microglia, and hippocampal neurons (45–49). Beyond humoral transmission, inflammatory signals can also penetrate the brain parenchyma through immune cell-mediated routes. For instance, circulating peripheral monocytes can traverse the BBB and differentiate into macrophages, thereby disrupting synaptic integrity and altering neuronal signaling. Ultimately, these cascading events culminate in neuroinflammation and neurocognitive deficits, which are characteristic hallmarks of cirrhosis and NASH-associated cognitive impairment. Robust experimental evidence corroborates this liver-brain inflammatory axis. For example, in murine models of NASH, the circulating hepatokine LCN2 crosses the BBB to activate microglia, inducing neuroinflammation that precipitates cognitive decline (50). Furthermore, targeted blockade of key inflammatory mediators can significantly alleviate these neuropathological alterations. Notably, treating cirrhotic rats with the TNF-α inhibitor etanercept not only restores spatial learning but also attenuates structural neuronal damage within the hippocampus (51) (**Figure 3**).

Primary bile acids are synthesized in the liver and secreted into the intestinal tract (**Table 1**). Within the terminal ileum, approximately 95% of these molecules are actively reabsorbed by the apical sodium-dependent bile acid transporter (ASBT/SLC10A2) and returned to the liver via the portal vein, thereby driving efficient enterohepatic circulation (52). The remaining 5% escapes reabsorption and passes into the colon, where commensal bacteria metabolize them into secondary bile acids, predominantly deoxycholic acid (DCA) and lithocholic acid (LCA). This biotransformation alters their physicochemical properties and confers novel biological functions (53). While a portion of these bile acids enters the systemic circulation, they extend beyond their classical enteric roles and cross the BBB via multiple pathways to modulate central neural activity. Highly lipophilic, unconjugated bile acids permeate the BBB through passive diffusion (54). Conversely, conjugated bile acids—due to their highly polar and charged nature—were long assumed to be excluded from BBB transit. However, recent studies have identified the expression of various organic anion-transporting polypeptides (OATPs) at the BBB, which facilitate the low-level, bidirectional transport of these conjugated species (55). Within the CNS, bile acids engage TGR5 (a G-protein-coupled receptor) and FXR (a nuclear receptor) expressed on neurons and glia. Activation of these receptors modulates microglial polarization, shifting the balance between neuroprotection and neurotoxicity. These pathways are highly relevant to the pathogenesis of hepatic encephalopathy, primary biliary cholangitis, and neurodegenerative diseases, prompting the clinical exploration of TGR5 agonists (e.g., INT-777) and FXR ligands as targeted therapies to modulate brain neuroinflammation (56–58) (**Figure 3**).

The primary source of systemic ammonia is the intestinal tract, where it exists in an equilibrium between gaseous NH_3_ and ionic NH_4_^+^. The intestinal microbiota generate substantial quantities of ammonia, primarily through the catabolism of nitrogenous substrates such as undigested proteins and amino acids. Under physiological conditions, this ammonia enters the liver via the portal vein and is almost entirely detoxified into urea via the urea cycle prior to renal excretion (59). However, in conditions such as cirrhosis or acute liver failure, severe hepatic dysfunction impairs urea synthesis. Concurrently, portosystemic shunts allow ammonia-rich portal venous blood to bypass hepatic clearance, directly entering the systemic circulation and inducing hyperammonemia (60, 61). During hepatic insufficiency, skeletal muscle emerges as a critical alternative site for ammonia detoxification via glutamine synthetase (GS)-mediated glutamine production (62). Consequently, reduced muscle mass (sarcopenia) diminishes this compensatory capacity, further exacerbating hyperammonemia.

Following intestinal absorption, xenobiotics—including therapeutic drugs—enter the liver via the portal vein and are predominantly metabolized by cytochrome P450 (CYP) enzymes, with only the unmetabolized fractions entering systemic circulation (63, 64) (**Table 1**). In pathological states such as non-alcoholic fatty liver disease (NAFLD) or cirrhosis, hepatic CYP expression and activity are significantly downregulated, resulting in diminished first-pass metabolism and prolonged systemic drug half-lives (65). This impaired clearance creates a synergistic neurotoxicity between ammonia and systemically circulating xenobiotics. Specifically, NH_3_ freely diffuses across the BBB, while NH_4_^+^ enters astrocytes via potassium channels and the Na^+^-K^+^-2Cl^-^ cotransporter (NKCC1). Within astrocytes—which highly express GS—ammonia is converted to glutamine, leading to massive glutamine accumulation, osmotic swelling, mitochondrial dysfunction, reactive oxygen species (ROS) production, and neurotransmitter dysregulation (66). Concurrently, circulating xenobiotics penetrate the BBB via passive diffusion or LBA-induced BBB hyperpermeability, directly interfering with neuronal receptor activity. These parallel mechanisms trigger widespread astrocyte swelling and the collapse of neurotransmitter homeostasis, ultimately culminating in hepatic encephalopathy (HE) and cirrhosis-related pharmacological encephalopathy. Clinically, managing these pathways involves administering lactulose and rifaximin to reduce intestinal ammonia production, alongside L-ornithine-L-aspartate to enhance ammonia detoxification in skeletal muscle (67, 68). Furthermore, for hepatically impaired patients, individualized drug dosing based on CYP450 activity and the potential application of BBB efflux transporter stimulators are highly recommended (69) (**Figure 3**).

Liver injury initiates a complex neuro-immune cascade that underpins the LBA—a dynamically integrated network of immune, humoral, and neural pathways. Activated by the altered hepatic metabolic-immune milieu, resident immune cells secrete pro-inflammatory cytokines, notably TNF-α and IL-1β. Acting as key local mediators via paracrine signaling, these cytokines do not need to cross the BBB; instead, they bind directly to IL-1R1 and TNFR on hepatic vagal afferent terminals, triggering neuronal depolarization. The specificity of this pathway is robustly demonstrated in IL-1R1 conditional knockout mice, which exhibit markedly diminished peripheral neural activation in response to IL-1β (70, 71). Once initiated, this neural conduit rapidly propagates peripheral inflammatory signals along the vagus nerve to the nucleus of the solitary tract (NTS) in the brainstem. The NTS subsequently relays these inputs to limbic centers, notably the amygdala, which critically mediate inflammation-associated anxiety-like behavior, depressive phenotypes, and sickness behavior (72, 73). Ultimately, rather than functioning as isolated conduits, these mutually reinforcing pathways tightly couple the peripheral hepatic state with central cognitive and affective processes. Clinically, modulating this pathological reflex may be achieved through targeted interventions such as vagus nerve stimulation (VNS) or local peripheral immune blockade (9, 74).

## 4 The role of the gut microbiota in the LBA signaling

**Figure 4:**
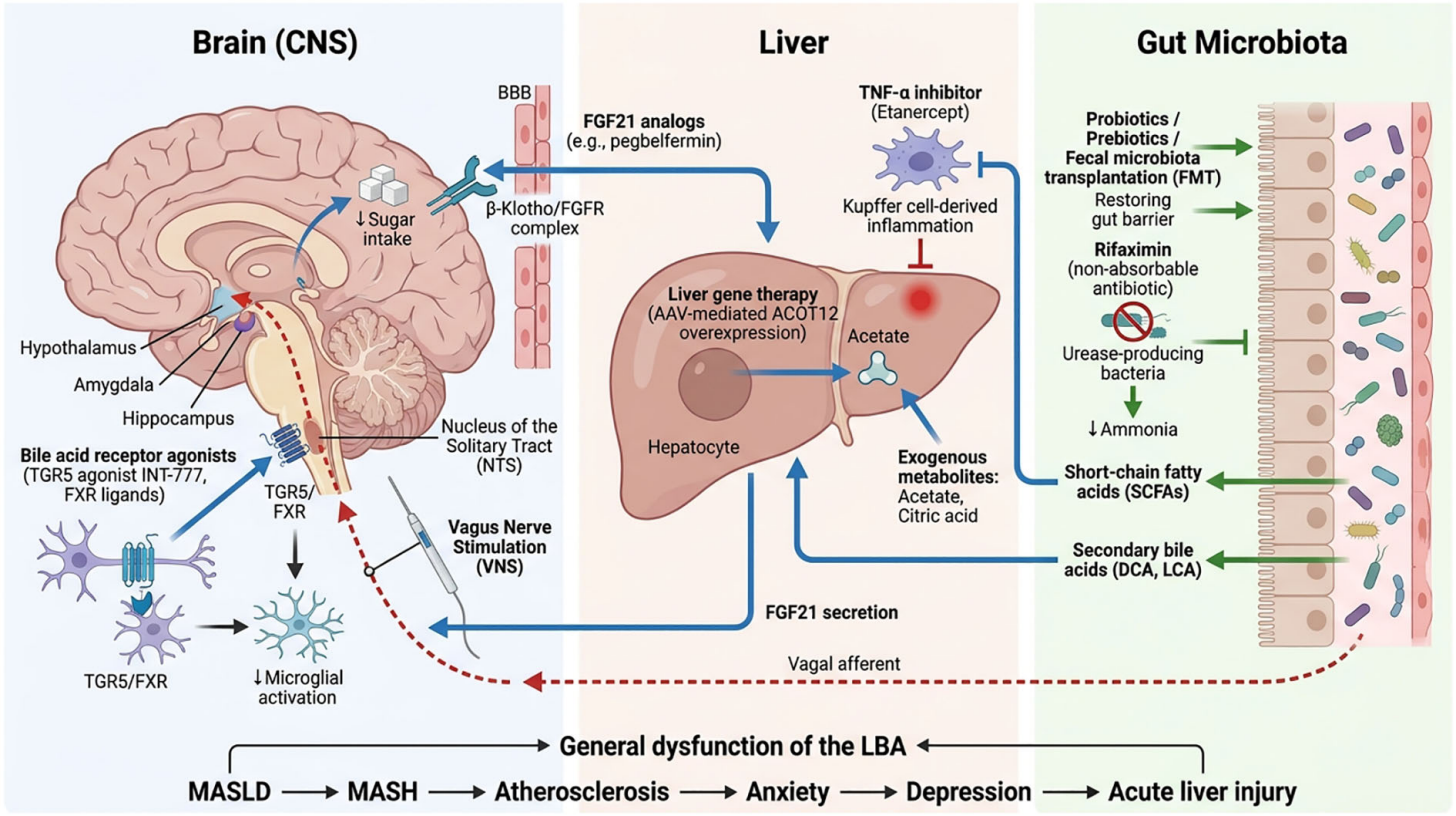
Therapeutic intervention strategies based on the liver-brain axis for multisystem diseases. This schematic illustrates the complex signaling crosstalk connecting the central nervous system (CNS), liver, and gut microbiota, highlighting targeted therapeutic strategies designed to ameliorate liver–brain axis (LBA) dysfunction. (1) CNS-targeted modulation: The hepatokine FGF21 and its analogs (e.g., pegbelfermin) cross the blood–brain barrier (BBB) to activate the β-Klotho/FGFR complex, thereby modulating feeding behavior and reducing sugar intake. Neuromodulation via vagus nerve stimulation (VNS) relays signals directly to the nucleus tractus solitarius (NTS). Furthermore, bile acid receptor agonists targeting TGR5 and FXR suppress microglial activation in critical brain regions, such as the hippocampus and amygdala. (2) Hepatic interventions: TNF-α inhibitors (e.g., etanercept) attenuate Kupffer cell-driven neuroinflammation and liver injury. Additionally, liver-directed gene therapy (e.g., AAV-mediated ACOT12 overexpression) restores hepatic metabolism by promoting the generation of protective metabolites, such as acetate. (3) Gut microbiota modulation: Interventions including probiotics, prebiotics, and fecal microbiota transplantation (FMT) restore intestinal barrier integrity. Concurrently, non-absorbable antibiotics (e.g., rifaximin) inhibit urease-producing bacteria, thereby lowering neurotoxic ammonia levels. Gut-derived metabolites—specifically short-chain fatty acids (SCFAs) and secondary bile acids (DCA, LCA)—exert prominent systemic effects via the circulation and vagal afferent pathways. In conclusion, restoring the homeostasis of this multi-organ axis establishes a comprehensive therapeutic framework for preventing and treating an interconnected continuum of LBA-associated pathologies, ranging from MASLD, MASH, and atherosclerosis to psychiatric disorders (e.g., anxiety, depression) and acute liver injury.

While the LBA delineates the direct bidirectional crosstalk between the liver and the CNS, this network is fundamentally modulated by the gut microbiome, thereby constituting a broader, highly integrated gut-liver-brain axis (75) (**Figure 4**).

Anatomically and physiologically, the liver serves as the primary firewall against intestinal metabolites entering via the portal circulation, establishing the gut microbiota as a critical upstream regulator of LBA signaling (76). As previously discussed, the generation and regulation of several key LBA signaling molecules are fundamentally microbiome-dependent. For instance, the biotransformation of liver-synthesized primary BAs into secondary BAs, such as deoxycholic acid (DCA) and lithocholic acid (LCA), is mediated by colonic commensal bacteria. These secondary BAs subsequently cross the BBB to modulate CNS neuroinflammation via FXR and TGR5 activation (77). Similarly, ammonia—the primary pathophysiological driver of hepatic encephalopathy—originates predominantly from the degradation of nitrogenous compounds by urease-producing gut microbiota. Under pathological conditions like cirrhosis, gut dysbiosis accelerates ammonia production; concurrently, portosystemic shunting and hepatic dysfunction allow this neurotoxin to bypass hepatic clearance, directly reaching the brain to induce astrocyte swelling (78, 79).

Furthermore, the gut microbiome profoundly influences LBA-driven neuroinflammation via gut-immune signaling. In chronic liver diseases, compromised intestinal barrier function—often termed “leaky gut”—permits the translocation of pathogen-associated molecular patterns (PAMPs, e.g., lipopolysaccharide (LPS)) and bacterial metabolites into the portal circulation. Upon reaching the liver, these microbial signals hyperactivate Kupffer cells, amplifying the hepatic release of pro-inflammatory cytokines (e.g., TNF-α, IL-1β, and IL-6) that ultimately propagate neuroinflammation to the brain (80). Beyond these immune cascades, the gut microbiota synthesizes potent neuroactive compounds, including short-chain fatty acids (SCFAs) and tryptophan metabolites, which enter the systemic circulation to directly modulate BBB integrity and microglial phenotype (81, 82) (**Figure 4**).

Consequently, conceptualizing the microbiome as a cornerstone of the LBA identifies critical upstream therapeutic targets and underscores the immense potential of interventions aimed at reshaping the gut microenvironment. Strategies such as non-absorbable antibiotics (e.g., rifaximin, which suppresses urease-producing bacteria) (83), probiotics, prebiotics, and fecal microbiota transplantation (FMT) have shown significant clinical promise (**Figure 4**). By mitigating microbial dysbiosis and restoring intestinal barrier integrity (84). these microbiome-targeted therapies effectively attenuate pathological liver-to-brain signaling, offering a robust therapeutic paradigm for LBA-associated neurocognitive and neuropsychiatric disorders.

## 5 Species differences of the LBA

The LBA represents a bidirectional communication network between the liver and the central nervous system, mediated by neural, humoral, and immune signaling pathways. While previous sections have extensively discussed various mechanisms and therapeutic targets, evaluating these findings requires a strict hierarchy of evidence. Notably, key translational differences between humans and mice are evident in the signaling molecules involved, the integrity and permeability of the BBB, and the composition of the hepatic and CNS immune microenvironments (**Table 2**).

**Table 2:** A summary of studies on species differences in humans and mice.

Key areas of species differences	Animal evidence	Human observational studies	Human mechanistic data	Interventional clinical evidence	Reference
BAs Profile	>95% taurine-bound; CYP2C70 → β/α-MCA (FXR antagonists)	Glycine:taurine ~3:1	Primary BAs: CA/CDCA (FXR agonists); no CYP2C70		(90–95)
BBB	Higher baseline permeability; sensitive to neuroinflammation/toxins	Baseline P-gp lower than rodents; ↑ with age	Claudin-5 ↑; tighter junctional arrays (postmortem)	High attrition rate	(96–107)
The hepatic immune microenvironment	iNKT cells: 30–50% of liver lymphocytes; central role	iNKT not primary drivers of inflammation	iNKT frequency <1%	Overestimation of clinical efficacy	(108, 109)
CNS Immune Microenvironment	Peripheral T cells/monocytes → extensive parenchymal infiltration	Infiltration kinetics: highly heterogeneous	Human microglia: distinct gene signature; P2RY12 ↓ upon challenge	Extremely limited; poor clinical conversion	(110–113)

First, within crucial signaling cascades, profound species-specific differences exist in BA chemistry (**Table 2**). BAs are synthesized in the liver from cholesterol via classic (CYP7A1-initiated) and alternative (CYP27A1-initiated) pathways, yielding the primary BAs cholic acid (CA) and chenodeoxycholic acid (CDCA) (85). Following biliary secretion, these are metabolized by the gut microbiota into secondary BAs, which collectively regulate metabolic and immune homeostasis by activating the nuclear receptor FXR and the membrane-bound receptor TGR5 (86, 87). Regarding conjugation, clinical and mechanistic studies demonstrate that human primary BAs are predominantly amidated with glycine (glycine-to-taurine ratio ~3:1), whereas murine BAs are almost exclusively taurine-conjugated (>95%) (88). The most fundamental divergence lies in the primary BA profile itself. While human hepatocytes predominantly produce CA and CDCA, murine hepatocytes express the unique enzyme CYP2C70, which catalyzes the 6β-hydroxylation of CDCA to generate α-muricholic acid (α-MCA) and its C7 epimer, β-muricholic acid (β-MCA) (89, 90). Functionally, CA and CDCA serve as potent FXR agonists, whereas β-MCA acts as a naturally occurring FXR antagonist. This biochemical disparity underscores a critical divergence in BA-mediated regulatory signaling (89). Moreover, interventional clinical evidence directly targeting these specific BA dichotomies within the LBA remains strikingly sparse, leaving a substantial gap between murine mechanisms and clinical reality.

Second, the molecular composition of the BBB exhibits significant interspecies variation, particularly regarding tight junction architecture and transporter expression profiles (91) (**Table 2**). Although Claudin-5—a critical member of the claudin family—is robustly expressed in the microvascular endothelial cells of both species, post-mortem analyses reveal that the human BBB endothelium possesses greater Claudin-5 abundance and more densely organized junctional arrays than its murine counterpart (92, 93). A more profound divergence exists within efflux transporter systems, most notably P-glycoprotein (P-gp), which mediates the active extrusion of lipophilic compounds from the brain. Observational studies indicate that baseline P-gp expression at the human BBB is substantially lower than in rodents, and its activity undergoes age-dependent upregulation (94, 95). Additional physiological distinctions encompass pericyte coverage, baseline metabolic rates, and the developmental trajectories of the neurovascular unit (NVU) (96–98). Collectively, these differences underpin the fundamentally distinct permeability profiles of human and rodent BBBs. Rodent BBBs generally exhibit higher baseline permeability, rendering them more susceptible to neuroinflammatory stimuli and exogenous toxins. While this experimental feature expedites phenotypic detection and target validation in preclinical models (99, 100). it systematically leads to the overestimation of CNS exposure to therapeutic agents in humans. Consequently, preclinical efficacies frequently fail to translate into successful interventional clinical outcomes, serving as a primary driver of the high attrition rates in neuropharmaceutical development (101, 102).

Third, the immune microenvironments of both the liver and the CNS exhibit profound species-specific disparities (**Table 2**). In the liver, the most striking divergence lies in the abundance of invariant natural killer T (iNKT) cells. Preclinical evidence indicates that iNKT cells constitute 30–50% of total hepatic lymphocytes in murine livers and are central to inflammatory modulation; conversely, human tissue analyses reveal their frequency is negligible, typically remaining below 1% (103, 104). This discrepancy introduces an inherent translational bias, frequently leading to a systematic overestimation of iNKT cell contributions to human liver disease.

Similarly, within the CNS, while the core transcriptomic signature of resident microglia is evolutionarily conserved, crucial divergences remain (**Table 2**). *In vitro* mechanistic studies reveal that human microglia express a distinct subset of genes absent in mice and demonstrate markedly altered baseline expression of otherwise conserved markers. Notably, upon inflammatory challenge, human microglia display heightened susceptibility to the downregulation of core homeostatic markers, such as P2RY12 (105, 106).

Furthermore, while *in vivo* murine studies frequently document significant infiltration of T cells and monocytes into the CNS parenchyma under pathological conditions, human observational data portray a highly restricted and heterogeneous infiltration kinetic, heavily constrained by the aforementioned human BBB architecture (107, 108). Consequently, murine models frequently overestimate the direct contribution of liver-derived peripheral immune cells to neuroinflammation, confounding the translation of immunomodulatory therapeutic strategies.

In summary, the LBA exhibits significant species-specific differences across metabolic, structural, and immunological domains. Bridging this translational gap and avoiding the erroneous extrapolation of murine data to human pathophysiology requires such an evidence-based synthesis.

## 6 Mechanisms associating the LBA and diseases

The LBA has emerged as a focal point in metabolic disease research, marking a paradigm shift from viewing diabetes and obesity as isolated peripheral disorders to recognizing them as systemic conditions driven by disrupted bidirectional communication between the liver and the CNS (**Figure 4**). By coordinating complex neuroendocrine and metabolic signaling networks, this axis mediates multiorgan dysfunction, inextricably linking hepatic perturbations to neuroinflammatory and behavioral outcomes. Consequently, elucidating the intricate mechanisms underlying this crosstalk is paramount for redefining the pathophysiological landscape of systemic metabolic disorders.

The LBA is fundamental to energy homeostasis, primarily mediated through the hypothalamic regulation of peripheral metabolism. Functioning as a central integrative hub, the hypothalamus processes circulating metabolic signals to orchestrate systemic glucose and energy balance (109). Under physiological conditions, distinct hypothalamic neuronal populations modulate hepatic glucose production and lipid metabolism via PI3K/Akt and STAT3 signaling pathways (110). The activation of these cascades effectively suppresses hepatic gluconeogenesis, enhances peripheral insulin sensitivity, curtails appetite, and promotes energy expenditure (111). Conversely, prolonged consumption of a high-fat diet (HFD) provokes the activation of hypothalamic microglia and astrocytes. This reactive gliosis precipitates localized neuroinflammation and the robust secretion of proinflammatory cytokines—specifically IL-1β and TNF-α—which collectively induce central leptin and insulin resistance. This refractory state blunts the hypothalamic capacity to accurately sense and integrate peripheral metabolic cues, thereby disrupting essential efferent regulatory signals to the liver. The ensuing sympathetic overdrive to hepatic tissue persistently drives unchecked gluconeogenesis and glycogenolysis despite elevated systemic glucose, culminating in fasting hyperglycemia. Ultimately, these central neuroinflammatory cascades and subsequent autonomic dysregulations serve as primary pathogenic drivers of obesity and its associated metabolic comorbidities (112, 113).

Rather than functioning merely as a passive recipient within the LBA, the liver actively orchestrates systemic metabolic regulation through the secretion of hepatokines (**Figure 4**). Among these, FGF21 remains the most extensively characterized. Under physiological conditions, the liver secretes FGF21 in response to metabolic stress; this hormone subsequently crosses the BBB to act upon glutamatergic neurons within the ventromedial hypothalamus (VMH), thereby suppressing hepatic glucose production and modulating energy balance (114, 115). However, in the context of obesity, the central actions of FGF21 are profoundly blunted despite markedly elevated circulating levels, indicating central FGF21 resistance. This resistance compromises central sensitivity to hepatic metabolic cues, precipitating dysregulated feeding behaviors and diminished energy expenditure, which collectively exacerbate central insulin resistance and neuroinflammation. Furthermore, whole-brain neuronal activation mapping in obese mice subjected to long-term HFD feeding has revealed that the capacity of exogenous FGF21 to activate key hypothalamic loci—most notably the paraventricular nucleus (PVN)—is significantly attenuated, providing direct neuroanatomical evidence of central resistance (115–117). Beyond endocrine signaling, the liver also operates as a real-time metabolic sensor. It continuously transmits its metabolic status via vagal sensory afferents to the nucleus of the solitary tract (NTS) in the brainstem, which subsequently relays these signals to higher-order integrative centers, including the hypothalamus (118). Beyond endocrine signaling, the liver operates as a real-time metabolic sensor, continuously transmitting its metabolic status via vagal sensory afferents to the nucleus of the solitary tract (NTS) in the brainstem, which relays these signals to higher-order integrative centers, including the hypothalamus (107). During obesity, the transmission of aberrant hepatic signals—driven by localized lipid accumulation and inflammation—via these vagal pathways disrupts central satiety networks, thereby promoting maladaptive hyperphagic behaviors (119).

As obesity and diabetes advance, LBA dysfunction evolves from isolated metabolic perturbations to widespread multisystemic injury. Cardiometabolic disorders represent the most prominent peripheral manifestations of this progression, encompassing atherosclerosis, heart failure, and metabolic dysfunction-associated steatotic liver disease (MASLD) (120). Concurrently, these peripheral metabolic derangements impact the CNS through systemic inflammatory mediators and compromised BBB integrity, fostering neuropsychiatric comorbidities such as anxiety and depression (121). In essence, this progressive, multiorgan pathology is fundamentally underpinned by the profound dysregulation of the LBA.

In the context of cardiometabolic diseases, LBA dysfunction accelerates the pathogenesis of atherosclerosis and heart failure (122). Specifically, systemic insulin resistance and autonomic sympathetic overdrive act synergistically to promote hepatic lipid accumulation, driving the onset and progression of MASLD (123). Amidst this metabolic derangement, the compensatory elevation of circulating FGF21 is accompanied by the robust secretion of hepatocyte-derived proinflammatory cytokines, notably TNF-α and IL-6 (124, 125). These circulating mediators directly compromise vascular endothelial integrity, perpetuating a state of chronic, low-grade systemic inflammation (126). Concurrently, this relentless metabolic inflammation disrupts the hypothalamic regulation of the autonomic nervous system. The resultant excessive sympathetic outflow not only elevates systemic blood pressure and cardiac afterload but also exacerbates hepatic gluconeogenesis and *de novo* lipogenesis. Thus, this bidirectional amplification between MASLD and cardiovascular pathology establishes a self-perpetuating pathogenic cycle (127, 128). Therefore, profound LBA dysregulation serves as the unifying pathophysiological substrate for the concurrent development of atherosclerosis, MASLD, and heart failure.

Crucially, the bidirectional nature of the LBA dictates that the CNS is not merely a unidirectional regulator of peripheral metabolism, but also a vulnerable target of peripheral pathologies. Under conditions of profound insulin resistance, hepatocyte-derived inflammatory mediators enter the systemic circulation and signal to the CNS via both circumventricular neurohumoral pathways and vagal sensory afferents. This crosstalk triggers the robust activation of microglia within key limbic and regulatory structures, including the hypothalamus, amygdala, and hippocampus (129). The ensuing neuroinflammatory cascade profoundly impairs synaptic plasticity and disrupts neurotransmitter metabolism. Collectively, these structural and functional CNS alterations constitute the core pathological mechanisms underlying the heightened prevalence of neuropsychiatric comorbidities, particularly anxiety and depressive disorders, frequently observed in patients with obesity and diabetes (130, 131).

## 7 The LBA, a new therapeutic target

The LBA has emerged as a compelling therapeutic target for metabolic, neurological, and cardiovascular disorders, reflecting a paradigm shift from organ-centric models toward a systems-level understanding of bidirectional crosstalk. This framework reconceptualizes the liver and CNS as dynamically interconnected and reciprocally regulated entities rather than isolated functional units (**Figure 4**). Accumulating evidence from diverse preclinical models underscores the substantial translational potential of modulating this axis. Specifically, interventions targeting neuroinflammatory cascades, hepatokine signaling, and autonomic neural circuits have demonstrated preliminary yet promising efficacy in restoring metabolic and behavioral homeostasis (**Figure 4**). Translating these findings to human pathology presents significant hurdles due to interspecies biological variations and a lack of clinical validation.

### 7.1 MASLD and MASH

MASLD is currently the most prevalent chronic liver disease worldwide, encompassing a clinical spectrum that ranges from isolated steatosis to its progressive form, while MASH is characterized by hepatic inflammation and hepatocyte injury (132). Epidemiological data indicate that MASLD affects approximately 30% of the global adult population, A comparable prevalence of 30.4% is observed in China, where an estimated 6.7% of cases progress to MASH (133). The disease burden is markedly disproportionate among high-risk demographics: men exhibit a 1.5- to 2-fold higher prevalence than women, and incidence rates climb sharply in individuals aged 50 years and older. Furthermore, MASLD prevalence escalates to 60–70% in patients with type 2 diabetes and frequently surpasses 70% in those with obesity (134). These epidemiological patterns underscore that MASLD is fundamentally the hepatic manifestation of metabolic syndrome and obesity, driving a bidirectional, self-perpetuating pathogenic loop. Consequently, while the liver-brain axis represents a compelling emerging framework for understanding MASLD pathophysiology, current clinical evidence does not yet support its status as the primary or most extensively exploited therapeutic target across this disease spectrum.

A recent study demonstrated that the selective ablation of liver-innervating vagal sensory neurons—achieved via a genetic approach combined with viral tracing—effectively disrupts the sensory afferent limb of the liver-brain axis (20). Following 10 to 12 weeks of HFD feeding, mice subjected to this targeted denervation exhibited significant weight loss and increased energy expenditure. Notably, hepatic steatosis and triglyceride accumulation were markedly attenuated, with near-complete prevention observed specifically in male cohorts (20). At the molecular level, the expression of key hepatic lipogenic genes, including *Srebp1c*, *Fasn*, and *Cd36*, was significantly downregulated, whereas transcripts associated with lipid oxidation were concomitantly upregulated. Furthermore, this sensory denervation ameliorated HFD-induced anxiety-like behaviors. Collectively, these findings provide compelling preclinical evidence that neuromodulatory interventions targeting the liver-brain axis hold significant therapeutic promise for the management of MASLD (20).

Building upon this foundation, recent studies demonstrate that targeting specific CNS neuronal circuits can profoundly ameliorate metabolic liver diseases (135). For instance, the administration of FGF21 in diet-induced models of MASH markedly attenuates disease severity (**Figure 4**). This is evidenced by robust reductions in hepatic steatosis, cholesterol accumulation, and circulating transaminase levels, alongside broader histological improvements (135). Mechanistically, these hepatoprotective effects require the activation of β-klotho (KLB)-positive glutamatergic neurons within the CNS and the subsequent recruitment of hepatic sympathetic efferent pathways. Strikingly, the therapeutic efficacy of FGF21 is entirely abolished following the neuron-specific deletion of *Klb*, establishing the CNS–liver sympathetic axis as indispensable for MASH reversal (135).

Collectively, current evidence indicates that therapeutic modulation of the liver–brain axis effectively reduces hepatic lipid accumulation and inflammatory responses in murine models of MASLD and MASH. Moreover, these interventions concurrently ameliorate associated neurobehavioral deficits, such as anxiety- and depression-like behaviors, thereby providing a robust rationale for axis-targeted therapies. However, these promising findings are currently restricted to rodent paradigms. Given the substantial interspecies variations between mice and humans—particularly regarding the neuroanatomical projections of the liver-brain axis, bile acid metabolomic profiles, and the pathophysiological trajectories of steatohepatitis—the clinical translation of these preclinical discoveries demands rigorous validation. Furthermore, the reproducibility of these interventions across independent laboratories remains to be systematically evaluated.

### 7.2 Anxiety and depression

Recent advances in liver-brain axis research have profoundly redefined our understanding of neuropsychiatric complications in chronic liver diseases. This paradigm shift redirects the field’s focus from the passive diffusion of neurotoxic metabolites to the active, bidirectional modulation of emotional states by liver-derived signaling molecules (15).

Utilizing a chronic social defeat stress (CSDS) murine model, recent studies demonstrate that excessive glucocorticoid signaling suppresses the hepatic transcription of *Acot12*—a thioesterase predominantly expressed in the liver—thereby depleting circulating acetate levels. Consequently, acetate has emerged as a pivotal metabolic messenger that regulates depression susceptibility via the liver-brain axis. Strikingly, liver-specific overexpression of *Acot12* using adeno-associated viral (AAV) vectors successfully restored plasma acetate concentrations and ameliorated depressive-like behaviors in CSDS-susceptible mice (**Figure 4**). Furthermore, systemic administration of exogenous acetate elicits rapid-onset antidepressant-like effects in murine models. Behavioral assessments reveal that this intervention significantly ameliorates depression-like phenotypes, evidenced by enhanced social interaction and diminished behavioral despair. Mechanistically, circulating acetate crosses the blood-brain barrier and accesses the ventral hippocampus, where it drives a region-specific increase in histone acetylation (136). This epigenetic modification subsequently upregulates astrocytic PD-L1 expression, which effectively suppresses microglia-mediated neuroinflammation and restores essential inhibitory synaptic transmission (136).

Collectively, these findings underscore that augmenting hepatic acetate bioavailability—either through targeted *Acot12* gene therapy or direct acetate supplementation—represents a promising novel therapeutic strategy for mitigating neuropsychiatric disorders via the peripheral modulation of central neuroinflammatory cascades. However, these findings are currently derived exclusively from the CSDS murine model (125) and lack validation in clinical depression cohorts. Furthermore, AAV-mediated overexpression of the *Acot12* gene in the liver poses inherent risks of long-term off-target effects and immunogenicity. Similarly, the optimal dosage, route of administration, and long-term safety profile of exogenous acetate supplementation require rigorous systematic evaluation before proceeding to clinical translation.

### 7.3 Atherosclerosis

Atherosclerosis is a chronic vascular disease characterized by the accumulation of lipid-rich fibrous plaques within the intima of large and medium arteries. Its pathogenesis is driven by the oxidative modification of low-density lipoprotein (LDL) particles, endothelial dysfunction, and inflammatory cell activation, ultimately culminating in luminal stenosis, plaque rupture, acute thrombosis, and ischemic organ injury. Although emerging evidence highlights atherosclerosis as a systemic disorder orchestrated by the interplay between dysregulated lipid metabolism and chronic inflammation (137), substantial residual cardiovascular risk persists despite optimal LDL cholesterol (LDL-C) reduction (138).

To address this residual risk, recent preclinical findings demonstrate that oral oxytocin administration attenuates atherosclerotic progression via modulation of the brain-liver axis. In a recent study, *Apoe*^-^/^-^ mice fed a HFD were subjected to either 12 weeks of social isolation—to simulate human psychosocial deprivation—or standard group housing. Chronic social isolation induced a marked decline in hypothalamic oxytocin synthesis. This central neuroendocrine deficiency subsequently disrupted peripheral hepatic lipid homeostasis via impaired oxytocin receptor (OXTR) signaling. Specifically, transcriptional suppression of *Cyp7a1*, alongside aberrant regulation of *Angptl4* and *Angptl8*, induced severe dyslipidemia and accelerated atherogenesis (139). Notably, continuous oral oxytocin supplementation effectively reversed these isolation-induced metabolic perturbations. In the treated cohort, serum lipid profiles normalized to levels observed in group-housed controls, concurrent with the restoration of hepatic *Cyp7a1* and *Angptl4/8* expression and systemic plasma lipoprotein lipase (LPL) activity (139). Ultimately, this neuro-metabolic intervention yielded significant macrovascular benefits, reducing overall aortic plaque burden by approximately 40%, enhancing plaque stability, and attenuating concurrent hepatic steatosis (139). Despite these promising pre-clinical findings, several limitations must be acknowledged. First, the reliance on a single genetic model (*Apoe*^-^/^-^ mice) fails to fully capture the complex polygenic and environmental etiology of human atherosclerosis. Furthermore, inherent species-specific differences in central and peripheral oxytocin regulatory networks between rodents and humans may complicate clinical translatability. Consequently, substantial data gaps remain regarding the pharmacokinetics, long-term safety, and therapeutic efficacy of oral oxytocin in patients with cardiovascular disease, necessitating further clinical investigation.

### 7.4 Acute liver injury

While traditional therapeutic paradigms for acute liver injury have predominantly focused on modulating local hepatic or systemic immune responses, neuromodulation has recently emerged as a promising alternative strategy targeting the brain-liver axis. Specifically, recent evidence demonstrates that sensory neuromodulation via sour taste stimulation attenuates macrophage-driven hepatic ischemia-reperfusion injury (HIRI). In a murine HIRI model, oral administration of citric acid (50 mM) significantly blunted serum alanine aminotransferase (ALT) and aspartate aminotransferase (AST) surges by 40–50% (140). Concurrently, this intervention suppressed the systemic release of pro-inflammatory cytokines, including IL-6 and TNF-α, and markedly reduced hepatocellular necrosis and apoptosis. Mechanistically, sour taste perception activates a specific gustatory-brain-vagal neural circuit that suppresses the neuronal expression and release of the chemokine-like protein TAFA2 (140). The subsequent decline in circulating TAFA2 prevents the activation of C-C chemokine receptor type 2 (CCR2) on peripheral macrophages, thereby abrogating their chemotactic infiltration into the hepatic parenchyma. Corroborating this mechanism, *Tafa2*^-^/^-^ mice exhibited an approximately 60% reduction in overall liver injury and a greater than 70% decrease in macrophage infiltration following HIRI (140).

Crucially, a pilot clinical trial involving patients undergoing hepatic resection translated these preclinical findings, revealing substantial therapeutic potential. Preoperative citric acid mouth rinsing yielded a significant 35% mean reduction in postoperative day 1 ALT levels compared to standard-of-care controls. Most notably, no patients in the intervention cohort developed severe acute liver injury (defined as ALT > 500 U/L), in stark contrast to 40% (12 of 30) of the control group (140). However, while these preliminary human data are encouraging, several limitations exist. The trial was single-center and small-scale—lacking a specified sample size for the intervention group—and failed to report potential adverse events associated with citric acid rinsing, such as oral mucosal irritation or enamel erosion. Therefore, the generalizability and safety of this intervention necessitate rigorous validation through larger, multicenter randomized controlled trials.

## 8 Conclusions and evaluation

Investigations into the LBA fundamentally challenge the classical paradigm of physiological compartmentalization by revealing a dynamic, bidirectional network linking the liver and the CNS. Emerging evidence establishes the liver as a central regulatory hub that integrates neural, humoral, and immune pathways to modulate CNS immunity, systemic metabolic homeostasis, and complex behavioral responses. This systemic crosstalk not only elucidates the shared pathophysiological underpinnings of neuropsychiatric and cardiometabolic diseases but also enables novel, peripherally targeted interventions (**Figure 4**).

Current LBA research is driven by several key themes: hyperammonemia-induced neurotoxicity in hepatic encephalopathy (141); functional remodeling of the BBB, which entails distinct substrate-specific transport systems—specifically, the altered influx of circulating bile acids predominantly mediated by the organic anion-transporting polypeptide (OATP) family (142); neuroimmune modulation mediated by hepatokines (e.g., fetuin-A, FGF21) and inflammatory cytokines (143); the integrative gut–liver–brain axis (28); and rapid autonomic neural circuits relaying signals from hepatic vagal afferents to the nucleus of the solitary tract (NTS) (144). Despite this rich mechanistic landscape, significant translational bottlenecks persist. The field’s overwhelming reliance on rodent models, which introduces pronounced species-specific disparities and temporal mismatches in disease progression—spanning metabolic enzyme kinetics, transporter profiles, microbiota compositions, and neuroinflammatory responses (145, 146). Moreover, prevailing reductionist, single-pathway approaches fail to capture the nonlinear interplay within the neurohumoral–immune–microbial network, resulting in a highly fragmented mechanistic map (147). Critically, a marked scarcity of prospective human mechanistic data and targeted clinical trials leaves the temporal causality of metabolic comorbidities largely unresolved (148). Furthermore, key biological variables—such as sex, age, and genetic heterogeneity—are systematically overlooked (149).

## 9 Prospect

To unlock the full therapeutic potential of the LBA, the field should make a transition from basic mechanistic discovery to rigorously designed clinical translation. Several strategic imperatives are urgently needed to achieve this. First, advanced platforms—such as human-derived organoids and organ-on-a-chip systems—must be developed to circumvent the inherent limitations of murine biology (150). Second, establishing longitudinal multi-omics cohorts that integrate liver function, neuroimaging, and cognitive phenotypes is crucial to clarifying causal relationships and temporal disease trajectories (151). Third, network medicine and machine learning should be leveraged to construct predictive models of multiorgan interactions (147). Finally, clinical trial designs must incorporate precision patient stratification based on hepatokine profiles, microbiome signatures, and genetic polymorphisms (152).

Most fundamentally, priority must be given to interventional trials that directly target LBA pathways—such as vagus nerve stimulation, selective antagonism of hepatokine receptors, or modulation of BBB transporters (153, 154)—rather than indirectly inferring LBA effects from trials confined solely to hepatic or psychiatric disorders. Although formidable translational barriers remain, particularly interspecies biological disparities, fully harnessing the LBA network promises to profoundly reshape our pathophysiological frameworks and clinical paradigms.

## Glossary

AAV: Adeno-associated virus
ACOT12: Acyl-CoA thioesterase 12
AD: Alzheimer’s disease
ALT: Alanine aminotransferase
Angptl4: Angiopoietin-like 4.
Angptl8: Angiopoietin-like 8
ASBT: Apical sodium-dependent bile acid transporter
AST: Aspartate aminotransferase
BBB: Blood-brain barrier
CA: Cholic acid
CCR2: C-C chemokine receptor type 2
CD36: Cluster of differentiation 36
CDCA: Chenodeoxycholic acid
CNS: Central nervous system
CSDS: Chronic social defeat stress
CYP: Cytochrome P450
DCA: Deoxycholic acid
Fasn: Fatty acid synthase
FGF21: Fibroblast growth factor 21
FXR: Farnesoid X receptor
GS: Glutamine synthetase
HE: Hepatic encephalopathy
HFD: High-fat diet
HIRI: Hepatic ischemia-reperfusion injury
IGF-1: Insulin-like growth factor 1
IL-1β: Interleukin-1 beta
IL-1R1: Interleukin-1 receptor type 1
IL-6: Interleukin-6
iNKT: Invariant natural killer T
KLB: Beta-klotho
LBA: Liver-brain axis
LCA: Lithocholic acid
LDL: Low-density lipoprotein
LDL-C: Low-density lipoprotein cholesterol
LPL: Lipoprotein lipase
MASH: Metabolic dysfunction-associated steatohepatitis
MASLD: Metabolic dysfunction-associated steatotic liver disease
NAFLD: Nonalcoholic fatty liver disease
NASH: Nonalcoholic steatohepatitis
NTS: Nucleus of the solitary tract
NVU: Neurovascular unit
OATPs: Organic anion-transporting polypeptides
OXTR: Oxytocin receptor
PD-L1: Programmed death-ligand 1
P-gp: P-glycoprotein
Srebp1c: Sterol regulatory element-binding protein 1c
STAT3: Signal transducer and activator of transcription 3
TAFA2: TAFA chemokine like protein 2
TGR5: Takeda G protein-coupled receptor 5
TNF-α: Tumor necrosis factor-alpha
VMH: Ventromedial hypothalamus
α-MCA: Alpha-muricholic acid
β-MCA SCFAs FMT: Beta-muricholic acid short-chain fatty acids fecal microbiota transplantation.

## References

[1] Sun H Saeedi P Karuranga S Pinkepank M Ogurtsova K Duncan BB . Idf diabetes atlas: global, regional and country-level diabetes prevalence estimates for 2021 and projections for 2045. Diabetes Res Clin Pract. (2022) 183:109119. doi: 10.1016/j.diabres.2021.109119 34879977 PMC11057359

[2] Kerr JA Patton GC Cini KI Abate YH Abbas N Abd Al Magied AHA . Global, regional, and national prevalence of child and adolescent overweight and obesity, 1990-2021, with forecasts to 2050: a forecasting study for the global burden of disease study 2021. Lancet. (2025) 405:785–812. doi: 10.1016/s0140-6736(25)00397-6 40049185 PMC11920006

[3] Chen P Zhang P Lin Z Qiu J Zou M Liu R . Association between changes in obesity status and neuropsychiatric health and brain structure in different glucose status. Front Nutr. (2025) 12:1676168. doi: 10.3389/fnut.2025.1676168 41112731 PMC12531048

[4] Biessels GJ Despa F . Cognitive decline and dementia in diabetes mellitus: mechanisms and clinical implications. Nat Rev Endocrinol. (2018) 14:591–604. doi: 10.1038/s41574-018-0048-7 30022099 PMC6397437

[5] Ahlawat A Walia V Garg M . Brain insulin resistance mediated cognitive impairment and neurodegeneration: type-3 diabetes or Alzheimer's disease. Acta Neurol Belg. (2025) 125:941–69. doi: 10.1007/s13760-024-02706-7 39762668

[6] Vegas-Suárez S Simón J Martínez-Chantar ML Moratalla R . Metabolic diffusion in neuropathologies: the relevance of brain-liver axis. Front Physiol. (2022) 13:864263. doi: 10.3389/fphys.2022.864263 35634148 PMC9134112

[7] Goel M Mittal A Jain VR Bharadwaj A Modi S Ahuja G . Integrative functions of the hypothalamus: linking cognition, emotion and physiology for well-being and adaptability. Ann Neurosci. (2025) 32:128–42. doi: 10.1177/09727531241255492 39544638 PMC11559822

[8] Yang X Qiu K Jiang Y Huang Y Zhang Y Liao Y . Metabolic crosstalk between liver and brain: from diseases to mechanisms. Int J Mol Sci. (2024) 25:7621. doi: 10.3390/ijms25147621 39062868 PMC11277155

[9] Zou J Li J Wang X Tang D Chen R . Neuroimmune modulation in liver pathophysiology. J Neuroinflamm. (2024) 21:188. doi: 10.1186/s12974-024-03181-w 39090741 PMC11295927

[10] Matsubara Y Kiyohara H Teratani T Mikami Y Kanai T . Organ and brain crosstalk: the liver-brain axis in gastrointestinal, liver, and pancreatic diseases. Neuropharmacology. (2022) 205:108915. doi: 10.1016/j.neuropharm.2021.108915 34919906

[11] Carata E Destino M Tenuzzo BA Panzarini E . Inter-organ crosstalk in neurodegenerative disease. Life (Basel). (2025) 15:1499. doi: 10.3390/life15101499 41157172 PMC12565391

[12] Sepehrinezhad A Zarifkar A Namvar G Shahbazi A Williams R . Astrocyte swelling in hepatic encephalopathy: molecular perspective of cytotoxic edema. Metab Brain Dis. (2020) 35:559–78. doi: 10.1007/s11011-020-00549-8 32146658

[13] Zhu R Liu L Zhang G Dong J Ren Z Li Z . The pathogenesis of gut microbiota in hepatic encephalopathy by the gut-liver-brain axis. Biosci Rep. (2023) 43:BSR20222524. doi: 10.1042/bsr20222524 37279097 PMC10272964

[14] Jayakumar AR Norenberg MD . Hyperammonemia in hepatic encephalopathy. J Clin Exp Hepatol. (2018) 8:272–80. doi: 10.1016/j.jceh.2018.06.007 30302044 PMC6175739

[15] Wang WT Xin YY Niu JQ Jin WL . The multiorgan crosstalk network of the liver-brain axis: from molecular mechanisms to emerging therapeutic targets. Mol Aspects Med. (2026) 108:101460. doi: 10.1016/j.mam.2026.101460 41679248

[16] Siddle M Gallego Durán R Goel D Renquist BJ Holt MK Hadjihambi A . Mechanistic insights into the liver-brain axis during chronic liver disease. Nat Rev Gastroenterol Hepatol. (2026) 23:166–88. doi: 10.1038/s41575-025-01142-z 41214287 PMC13034676

[17] Silva A Caron A . Pathophysiological mechanisms that alter the autonomic brain-liver communication in metabolic diseases. Endocrinology. (2021) 162:bqab164. doi: 10.1210/endocr/bqab164 34388249 PMC8455344

[18] Carobi C Magni F . The afferent innervation of the liver: a horseradish peroxidase study in the rat. Neurosci Lett. (1981) 23:269–74. doi: 10.1016/0304-3940(81)90009-4 6167912

[19] Berthoud HR Kressel M Neuhuber WL . An anterograde tracing study of the vagal innervation of rat liver, portal vein and biliary system. Anat Embryol (Berl). (1992) 186:431–42. doi: 10.1007/bf00185458 1280009

[20] Hwang J Lee S Okada J Liu L Pessin JE Chua SC . Liver-innervating vagal sensory neurons are indispensable for the development of hepatic steatosis and anxiety-like behavior in diet-induced obese mice. Nat Commun. (2025) 16:991. doi: 10.1038/s41467-025-56328-5 39856118 PMC11759694

[21] Ricardo JA Koh ET . Anatomical evidence of direct projections from the nucleus of the solitary tract to the hypothalamus, amygdala, and other forebrain structures in the rat. Brain Res. (1978) 153:1–26. doi: 10.1016/0006-8993(78)91125-3 679038

[22] Luo XY Ying SQ Cao Y Jin Y Jin F Zheng CX . Liver-based inter-organ communication: a disease perspective. Life Sci. (2024) 351:122824. doi: 10.1016/j.lfs.2024.122824 38862061

[23] Giovannoni L Pierzchala K De Roo M Braissant O Bruce S McLin VA . Effects of cholestasis and hyperammonemia on dendritic spine density and turnover in rat hippocampal neurons. Sci Rep. (2024) 14:29841. doi: 10.1038/s41598-024-80871-8 39617839 PMC11609291

[24] Zheng X Yang H Qin L Wang S Xie L Yang L . Bile duct ligation upregulates expression and function of L-amino acid transporter 1 at blood-brain barrier of rats via activation of aryl hydrocarbon receptor by bilirubin. Biomedicines. (2021) 9:1320. doi: 10.3390/biomedicines9101320 34680437 PMC8533316

[25] Banks WA . The blood-brain barrier as an endocrine tissue. Nat Rev Endocrinol. (2019) 15:444–55. doi: 10.1038/s41574-019-0213-7 31127254

[26] Kullmann S Kleinridders A Small DM Fritsche A Häring HU Preissl H . Central nervous pathways of insulin action in the control of metabolism and food intake. Lancet Diabetes Endocrinol. (2020) 8:524–34. doi: 10.1016/s2213-8587(20)30113-3 32445739

[27] D'Mello C Swain MG . Liver-brain inflammation axis. Am J Physiol Gastrointest Liver Physiol. (2011) 301:G749–61. doi: 10.1152/ajpgi.00184.2011 21868631

[28] D'Mello C Swain MG . Liver-brain interactions in inflammatory liver diseases: implications for fatigue and mood disorders. Brain Behav Immun. (2014) 35:9–20. doi: 10.1016/j.bbi.2013.10.009 24140301

[29] Giovanini L Wanionok N Perello M Cornejo MP . Brain-acting hepatokines: its impact on energy balance and metabolism. Front Neurosci. (2025) 19:1589110. doi: 10.3389/fnins.2025.1589110 40443802 PMC12119552

[30] Bono BS Koziel Ly NK Miller PA Williams-Ikhenoba J Dumiaty Y Chee MJ . Spatial distribution of beta-klotho mrna in the mouse hypothalamus, hippocampal region, subiculum, and amygdala. J Comp Neurol. (2022) 530:1634–57. doi: 10.1002/cne.25306 35143049

[31] Hultman K Scarlett JM Baquero AF Cornea A Zhang Y Salinas CBG . The central fibroblast growth factor receptor/beta klotho system: comprehensive mapping in mus musculus and comparisons to nonhuman primate and human samples using an automated in situ hybridization platform. J Comp Neurol. (2019) 527:2069–85. doi: 10.1002/cne.24668 30809795 PMC6546511

[32] Talukdar S Owen BM Song P Hernandez G Zhang Y Zhou Y . Fgf21 regulates sweet and alcohol preference. Cell Metab. (2016) 23:344–9. doi: 10.1016/j.cmet.2015.12.008 26724861 PMC4749404

[33] Jensen-Cody SO Flippo KH Claflin KE Yavuz Y Sapouckey SA Walters GC . Fgf21 signals to glutamatergic neurons in the ventromedial hypothalamus to suppress carbohydrate intake. Cell Metab. (2020) 32:273–286.e6. doi: 10.1016/j.cmet.2020.06.008 32640184 PMC7734879

[34] Solon-Biet SM Clark X Bell-Anderson K Rusu PM Perks R Freire T . Toward reconciling the roles of fgf21 in protein appetite, sweet preference, and energy expenditure. Cell Rep. (2023) 42:113536. doi: 10.1016/j.celrep.2023.113536 38060447

[35] Hill CM Laeger T Dehner M Albarado DC Clarke B Wanders D . Fgf21 signals protein status to the brain and adaptively regulates food choice and metabolism. Cell Rep. (2019) 27:2934–2947.e3. doi: 10.1016/j.celrep.2019.05.022 31167139 PMC6579533

[36] Gong P Zou Y Zhang W Tian Q Han S Xu Z . The neuroprotective effects of insulin-like growth factor 1 via the hippo/yap signaling pathway are mediated by the pi3k/akt cascade following cerebral ischemia/reperfusion injury. Brain Res Bull. (2021) 177:373–87. doi: 10.1016/j.brainresbull.2021.10.017 34717965

[37] Lin KN Zhang K Zhao W Huang SY Li H . Insulin-like growth factor 1 promotes cell proliferation by downregulation of g-protein-coupled receptor 17 expression via pi3k/akt/foxo1 signaling in sk-n-sh cells. Int J Mol Sci. (2022) 23:1513. doi: 10.3390/ijms23031513 35163437 PMC8835821

[38] Nishijima T Piriz J Duflot S Fernandez AM Gaitan G Gomez-Pinedo U . Neuronal activity drives localized blood-brain-barrier transport of serum insulin-like growth factor-I into the cns. Neuron. (2010) 67:834–46. doi: 10.1016/j.neuron.2010.08.007 20826314

[39] Joseph D'Ercole A Ye P . Expanding the mind: insulin-like growth factor I and brain development. Endocrinology. (2008) 149:5958–62. doi: 10.1210/en.2008-0920 18687773 PMC2613055

[40] Hayes CA Wilson D De Leon MA Mustapha MJ Morales S Odden MC . Insulin-like growth factor-1 and cognitive health: exploring cellular, preclinical, and clinical dimensions. Front Neuroendocrinol. (2025) 76:101161. doi: 10.1016/j.yfrne.2024.101161 39536910

[41] Kaur N Aran KR . Uncovering the intricacies of igf-1 in Alzheimer's disease: new insights from regulation to therapeutic targeting. Inflammopharmacology. (2025) 33:1311–30. doi: 10.1007/s10787-025-01641-0 39883327

[42] Carro E Trejo JL Gomez-Isla T LeRoith D Torres-Aleman I . Serum insulin-like growth factor I regulates brain amyloid-beta levels. Nat Med. (2002) 8:1390–7. doi: 10.1038/nm1202-793 12415260

[43] Yang D Song Y Yu S Ma Y Du W . Rational design of dual-targeting novel gpe-derived oligopeptide conjugates for Alzheimer's disease: synergistic inhibition of excitotoxicity and oxidative stress. ACS Chem Neurosci. (2026) 17:778–90. doi: 10.1021/acschemneuro.5c00871 41641951

[44] Hanson LR Frey WH . Intranasal delivery bypasses the blood-brain barrier to target therapeutic agents to the central nervous system and treat neurodegenerative disease. BMC Neurosci. (2008) 9 Suppl 3:S5. doi: 10.1186/1471-2202-9-s3-s5 19091002 PMC2604883

[45] Kaltenmeier C Wang R Popp B Geller D Tohme S Yazdani HO . Role of immuno-inflammatory signals in liver ischemia-reperfusion injury. Cells. (2022) 11:2222. doi: 10.3390/cells11142222 35883665 PMC9323912

[46] Schmidt-Arras D Rose-John S . Il-6 pathway in the liver: from physiopathology to therapy. J Hepatol. (2016) 64:1403–15. doi: 10.1016/j.jhep.2016.02.004 26867490

[47] Nguyen HH Swain MG . Avenues within the gut-liver-brain axis linking chronic liver disease and symptoms. Front Neurosci. (2023) 17:1171253. doi: 10.3389/fnins.2023.1171253 37521690 PMC10372440

[48] Gonçalves RA De Felice FG . The crosstalk between brain and periphery: implications for brain health and disease. Neuropharmacology. (2021) 197:108728. doi: 10.1016/j.neuropharm.2021.108728 34331960

[49] Banks WA . The blood-brain barrier in neuroimmunology: tales of separation and assimilation. Brain Behav Immun. (2015) 44:1–8. doi: 10.1016/j.bbi.2014.08.007 25172555 PMC4275374

[50] Mondal A Bose D Saha P Sarkar S Seth R Kimono D . Lipocalin 2 induces neuroinflammation and blood-brain barrier dysfunction through liver-brain axis in murine model of nonalcoholic steatohepatitis. J Neuroinflamm. (2020) 17:201. doi: 10.1186/s12974-020-01876-4 32622362 PMC7335438

[51] Nady R Ahmed RR Moustafa N Abdul-Hamid M . Tnf-α blockage by etanercept restores spatial learning and reduces cellular degeneration in the hippocampus during liver cirrhosis. Tissue Cell. (2023) 85:102249. doi: 10.1016/j.tice.2023.102249 37865039

[52] Hou Y Zhai X Wang X Wu Y Wang H Qin Y . Research progress on the relationship between bile acid metabolism and type 2 diabetes mellitus. Diabetol Metab Syndr. (2023) 15:235. doi: 10.1186/s13098-023-01207-6 37978556 PMC10656899

[53] Di Ciaula A Bonfrate L Khalil M Portincasa P . The interaction of bile acids and gut inflammation influences the pathogenesis of inflammatory bowel disease. Intern Emerg Med. (2023) 18:2181–97. doi: 10.1007/s11739-023-03343-3 37515676 PMC10635993

[54] Xing C Huang X Wang D Yu D Hou S Cui H . Roles of bile acids signaling in neuromodulation under physiological and pathological conditions. Cell Biosci. (2023) 13:106. doi: 10.1186/s13578-023-01053-z 37308953 PMC10258966

[55] Schäfer AM Meyer Zu Schwabedissen HE Grube M . Expression and function of organic anion transporting polypeptides in the human brain: physiological and pharmacological implications. Pharmaceutics. (2021) 13:834. doi: 10.3390/pharmaceutics13060834 34199715 PMC8226904

[56] Hu X Yan J Huang L Araujo C Peng J Gao L . Int-777 attenuates Nlrp3-Asc inflammasome-mediated neuroinflammation via Tgr5/cAMP/PKA signaling pathway after subarachnoid hemorrhage in rats. Brain Behav Immun. (2021) 91:587–600. doi: 10.1016/j.bbi.2020.09.016 32961266 PMC7749833

[57] Wu Y Qiu Y Su M Wang L Gong Q Wei X . Activation of the bile acid receptors Tgr5 and Fxr in the spinal dorsal horn alleviates neuropathic pain. CNS Neurosci Ther. (2023) 29:1981–98. doi: 10.1111/cns.14154 36880297 PMC10324360

[58] Ferrell JM Chiang JYL . Bile acid receptors and signaling crosstalk in the liver, gut and brain. Liver Res. (2021) 5:105–18. doi: 10.1016/j.livres.2021.07.002 39957847 PMC11791822

[59] Gallego-Durán R Hadjihambi A Ampuero J Rose CF Jalan R Romero-Gómez M . Ammonia-induced stress response in liver disease progression and hepatic encephalopathy. Nat Rev Gastroenterol Hepatol. (2024) 21:774–91. doi: 10.1038/s41575-024-00970-9 39251708

[60] Levitt DG Levitt MD . A model of blood-ammonia homeostasis based on a quantitative analysis of nitrogen metabolism in the multiple organs involved in the production, catabolism, and excretion of ammonia in humans. Clin Exp Gastroenterol. (2018) 11:193–215. doi: 10.2147/ceg.S160921 29872332 PMC5973424

[61] Meier C Burns K Manolikos C Fatovich D Bell DA . Hyperammonaemia: review of the pathophysiology, aetiology and investigation. Pathology. (2024) 56:763–72. doi: 10.1016/j.pathol.2024.06.002 39127541

[62] Chatauret N Desjardins P Zwingmann C Rose C Rao KV Butterworth RF . Direct molecular and spectroscopic evidence for increased ammonia removal capacity of skeletal muscle in acute liver failure. J Hepatol. (2006) 44:1083–8. doi: 10.1016/j.jhep.2005.11.048 16530878

[63] Xie F Ding X Zhang QY . An update on the role of intestinal cytochrome P450 enzymes in drug disposition. Acta Pharm Sin B. (2016) 6:374–83. doi: 10.1016/j.apsb.2016.07.012 27709006 PMC5045550

[64] Almazroo OA Miah MK Venkataramanan R . Drug metabolism in the liver. Clinics Liver Dis. (2017) 21:1–20. doi: 10.1016/j.cld.2016.08.001 27842765

[65] Zhu Y Chen L He Y Qin L Tan D Bai Z . The alteration of drug metabolism enzymes and pharmacokinetic parameters in nonalcoholic fatty liver disease: current animal models and clinical practice. Drug Metab Rev. (2023) 55:163–80. doi: 10.1080/03602532.2023.2202359 37042420

[66] Lu K . Cellular pathogenesis of hepatic encephalopathy: an update. Biomolecules. (2023) 13:396. doi: 10.3390/biom13020396 36830765 PMC9953810

[67] Oriko DO Khawaj Z Cheema MU Talreja A Tayyab MA Zamir MH . Therapeutic duel of rifaximin versus lactulose in hepatic encephalopathy: a systematic review. Cureus. (2025) 17:e86193. doi: 10.7759/cureus.86193 40677427 PMC12268772

[68] Zhang H Fu Y Lin M Nan Z Zhao D . Efficacy and safety of L-ornithine L-aspartate combined with lactulose in treatment of hepatic encephalopathy: a systematic review and meta-analysis of randomized controlled trial. Front Med (Lausanne). (2025) 12:1581792. doi: 10.3389/fmed.2025.1581792 40370740 PMC12075176

[69] Armani S Geier A Forst T Merle U Alpers DH Lunnon MW . Effect of changes in metabolic enzymes and transporters on drug metabolism in the context of liver disease: impact on pharmacokinetics and drug-drug interactions. Br J Clin Pharmacol. (2024) 90:942–58. doi: 10.1111/bcp.15990 38148609

[70] Xu GX Wei S Yu C Zhao SQ Yang WJ Feng YH . Activation of Kupffer cells in NAFLD and NASH: mechanisms and therapeutic interventions. Front Cell Dev Biol. (2023) 11:1199519. doi: 10.3389/fcell.2023.1199519 37261074 PMC10228659

[71] Niijima A . The afferent discharges from sensors for interleukin 1 beta in the hepatoportal system in the anesthetized rat. J Auton Nerv Syst. (1996) 61:287–91. doi: 10.1016/s0165-1838(96)00098-7 8988487

[72] Hu P Lu Y Pan BX Zhang WH . New insights into the pivotal role of the amygdala in inflammation-related depression and anxiety disorder. Int J Mol Sci. (2022) 23:11076. doi: 10.3390/ijms231911076 36232376 PMC9570160

[73] Berthoud HR Neuhuber WL . Functional and chemical anatomy of the afferent vagal system. Auton Neurosci. (2000) 85:1–17. doi: 10.1016/s1566-0702(00)00215-0 11189015

[74] Singh P Chaudhary M Kazmi JS Kuschner CE Volpe BT Chaudhuri TD . Vagus nerve stimulation: a targeted approach for reducing tissue-specific ischemic reperfusion injury. BioMed Pharmacother. (2025) 184:117898. doi: 10.1016/j.biopha.2025.117898 39923406

[75] Wu H Zhang Y Yu J Shi M . Editorial: gut-liver-brain axis: a complex network influences human health and diseases. Front Neurosci. (2023) 17:1241069. doi: 10.3389/fnins.2023.1241069 37521705 PMC10374336

[76] Aghara H Patel M Chadha P Parwani K Chaturvedi R Mandal P . Unraveling the gut-liver-brain axis: microbiome, inflammation, and emerging therapeutic approaches. Mediators Inflammation. (2025) 2025:6733477. doi: 10.1155/mi/6733477 40568349 PMC12197523

[77] Lee B Lee SM Song JW Choi JW . Gut microbiota metabolite messengers in brain function and pathology at a view of cell type-based receptor and enzyme reaction. Biomol Ther (Seoul). (2024) 32:403–23. doi: 10.4062/biomolther.2024.009 38898687 PMC11214962

[78] Liu J Xu Y Jiang B . Novel insights into pathogenesis and therapeutic strategies of hepatic encephalopathy, from the gut microbiota perspective. Front Cell Infect Microbiol. (2021) 11:586427. doi: 10.3389/fcimb.2021.586427 33692964 PMC7937792

[79] Vargas-Beltran AM Mialma-Omana SJ Vivanco-Tellez DO . Targeting gut microbiota in liver disease: a pharmacological approach for hepatic encephalopathy and beyond. World J Gastrointest Pharmacol Ther. (2025) 16:110271. doi: 10.4292/wjgpt.v16.i4.110271 41378067 PMC12687007

[80] Seo YS Shah VH . The role of gut-liver axis in the pathogenesis of liver cirrhosis and portal hypertension. Clin Mol Hepatol. (2012) 18:337–46. doi: 10.3350/cmh.2012.18.4.337 23323248 PMC3540369

[81] Cao Q Shen M Li R Liu Y Zeng Z Zhou J . Elucidating the specific mechanisms of the gut-brain axis: the short-chain fatty acids-microglia pathway. J Neuroinflamm. (2025) 22:133. doi: 10.1186/s12974-025-03454-y 40400035 PMC12093714

[82] Xia HH Huang JZ . Tryptophan metabolism at the crossroads of the neuro-immuno-microbial axis: implications for precision medicine in chronic diseases. Front Cell Infect Microbiol. (2025) 15:1707850. doi: 10.3389/fcimb.2025.1707850 41602107 PMC12833013

[83] Aggarwal N Shen H Lee LT Zhou L Zhu MT Koh XQ . Engineered commensals for metabolic modulation of the gut-liver-brain axis. Cell. (2026) S0092-8674(26)00384-3. doi: 10.1016/j.cell.2026.03.048 42034052

[84] Singh LS Singha LS Singh WS Singh YR Marak GK . Microbiome modulation as a therapeutic strategy for alcohol-induced gut dysbiosis and associated disorders. Antonie van Leeuwenhoek. (2025) 118:182. doi: 10.1007/s10482-025-02196-4 41160105

[85] Chiang JYL Ferrell JM . Up to date on cholesterol 7 alpha-hydroxylase (Cyp7a1) in bile acid synthesis. Liver Res. (2020) 4:47–63. doi: 10.1016/j.livres.2020.05.001 34290896 PMC8291349

[86] Chen F Gong L . Bile acid-microbiota interactions in cardiometabolic diseases: mechanisms and emerging therapeutic approaches. Front Microbiol. (2025) 16:1689026. doi: 10.3389/fmicb.2025.1689026 41480102 PMC12753514

[87] Yan W Zhang K Guo J Xu L . Bile acid-mediated gut-liver axis crosstalk: the role of nuclear receptor signaling in dynamic regulation of inflammatory networks. Front Immunol. (2025) 16:1595486. doi: 10.3389/fimmu.2025.1595486 40458398 PMC12127205

[88] Zheng D Ge K Qu C Sun T Wang J Jia W . Comparative profiling of serum, urine, and feces bile acids in humans, rats, and mice. Commun Biol. (2024) 7:641. doi: 10.1038/s42003-024-06321-3 38802554 PMC11130135

[89] Straniero S Laskar A Savva C Härdfeldt J Angelin B Rudling M . Of mice and men: murine bile acids explain species differences in the regulation of bile acid and cholesterol metabolism. J Lipid Res. (2020) 61:480–91. doi: 10.1194/jlr.RA119000307 32086245 PMC7112145

[90] Duan Z Yang T Li L Wang X Wei C Xia Z . Comparison of bile acids profiles in the enterohepatic circulation system of mice and rats. J Steroid Biochem Mol Biol. (2022) 220:106100. doi: 10.1016/j.jsbmb.2022.106100 35341917

[91] Kumabe H Masuda T Ito S Furihata T Toda A Mogi M . Proteome profile differences among human, monkey, and mouse brain microvessels and cultured brain microvascular endothelial cells. Fluids Barriers CNS. (2025) 22:53. doi: 10.1186/s12987-025-00650-z 40448242 PMC12124085

[92] Berndt P Winkler L Cording J Breitkreuz-Korff O Rex A Dithmer S . Tight junction proteins at the blood-brain barrier: far more than claudin-5. Cell Mol Life Sci. (2019) 76:1987–2002. doi: 10.1007/s00018-019-03030-7 30734065 PMC11105330

[93] Fontijn RD Rohlena J van Marle J Pannekoek H Horrevoets AJ . Limited contribution of claudin-5-dependent tight junction strands to endothelial barrier function. Eur J Cell Biol. (2006) 85:1131–44. doi: 10.1016/j.ejcb.2006.07.005 16959372

[94] Verscheijden LFM Koenderink JB de Wildt SN Russel FGM . Differences in P-glycoprotein activity in human and rodent blood-brain barrier assessed by mechanistic modelling. Arch Toxicol. (2021) 95:3015–29. doi: 10.1007/s00204-021-03115-y 34268580 PMC8380243

[95] Brenner SS Klotz U . P-glycoprotein function in the elderly. Eur J Clin Pharmacol. (2004) 60:97–102. doi: 10.1007/s00228-004-0733-4 15022031

[96] Bohannon DG Long D Kim WK . Understanding the heterogeneity of human pericyte subsets in blood-brain barrier homeostasis and neurological diseases. Cells. (2021) 10:890. doi: 10.3390/cells10040890 33919664 PMC8069782

[97] Herculano-Houzel S . Scaling of brain metabolism with a fixed energy budget per neuron: implications for neuronal activity, plasticity and evolution. PloS One. (2011) 6:e17514. doi: 10.1371/journal.pone.0017514 21390261 PMC3046985

[98] Saunders NR Dziegielewska KM Møllgård K Habgood MD . Physiology and molecular biology of barrier mechanisms in the fetal and neonatal brain. J Physiol. (2018) 596:5723–56. doi: 10.1113/jp275376 29774535 PMC6265560

[99] Uchida Y Yagi Y Takao M Tano M Umetsu M Hirano S . Comparison of absolute protein abundances of transporters and receptors among blood-brain barriers at different cerebral regions and the blood-spinal cord barrier in humans and rats. Mol Pharm. (2020) 17:2006–20. doi: 10.1021/acs.molpharmaceut.0c00178 32310660

[100] Hoshi Y Uchida Y Tachikawa M Inoue T Ohtsuki S Terasaki T . Quantitative atlas of blood-brain barrier transporters, receptors, and tight junction proteins in rats and common marmoset. J Pharm Sci. (2013) 102:3343–55. doi: 10.1002/jps.23575 23650139

[101] Deo AK Theil FP Nicolas JM . Confounding parameters in preclinical assessment of blood-brain barrier permeation: An overview with emphasis on species differences and effect of disease states. Mol Pharm. (2013) 10:1581–95. doi: 10.1021/mp300570z 23256608

[102] Strazielle N Blondel S Confais J El Khoury R Contamin H Ghersi-Egea JF . Molecular determinants of neuroprotection in blood-brain interfaces of the cynomolgus monkey. Front Pharmacol. (2025) 16:1523819. doi: 10.3389/fphar.2025.1523819 40144668 PMC11936797

[103] Gao B Radaeva S Park O . Liver natural killer and natural killer T cells: Immunobiology and emerging roles in liver diseases. J Leukoc Biol. (2009) 86:513–28. doi: 10.1189/JLB.0309135 19542050 PMC2735282

[104] Kenna T Golden-Mason L Porcelli SA Koezuka Y Hegarty JE O'Farrelly C . Nkt cells from normal and tumor-bearing human livers are phenotypically and functionally distinct from murine Nkt cells. J Immunol. (2003) 171:1775–9. doi: 10.4049/jimmunol.171.4.1775 12902477

[105] Galatro TF Holtman IR Lerario AM Vainchtein ID Brouwer N Sola PR . Transcriptomic analysis of purified human cortical microglia reveals age-associated changes. Nat Neurosci. (2017) 20:1162–71. doi: 10.1038/nn.4597 28671693

[106] van Wageningen TA Vlaar E Kooij G Jongenelen CAM Geurts JJG van Dam AM . Regulation of microglial Tmem119 and P2ry12 immunoreactivity in multiple sclerosis white and grey matter lesions is dependent on their inflammatory environment. Acta Neuropathol Commun. (2019) 7:206. doi: 10.1186/s40478-019-0850-z 31829283 PMC6907356

[107] Beuker C Strecker JK Rawal R Schmidt-Pogoda A Ruck T Wiendl H . Immune cell infiltration into the brain after ischemic stroke in humans compared to mice and rats: A systematic review and meta-analysis. Transl Stroke Res. (2021) 12:976–90. doi: 10.1007/s12975-021-00887-4 33496918 PMC8557159

[108] Healy LM Yaqubi M Ludwin S Antel JP . Species differences in immune-mediated CNS tissue injury and repair: A (neuro)inflammatory topic. Glia. (2020) 68:811–29. doi: 10.1002/glia.23746 31724770

[109] Inoue H . Central insulin-mediated regulation of hepatic glucose production [review. Endocr J. (2016) 63:1–7. doi: 10.1507/endocrj.EJ15-0540 26447084

[110] Ramnanan CJ Saraswathi V Smith MS Donahue EP Farmer B Farmer TD . Brain insulin action augments hepatic glycogen synthesis without suppressing glucose production or gluconeogenesis in dogs. J Clin Invest. (2011) 121:3713–23. doi: 10.1172/jci45472 21865644 PMC3163950

[111] Zhuang S Liu S Li R Duan H . Electroacupuncture alleviates insulin resistance and impacts the hypothalamic Irs-1/Pi3k/Akt pathway and Mirna-29a-3p in a rat model of type 2 diabetes. Acupunct Med. (2025) 43:104–13. doi: 10.1177/09645284251327205 40116412

[112] Rahman HH Bhusal A Lee WH Lee IK Suk K . Hypothalamic inflammation and malfunctioning glia in the pathophysiology of obesity and diabetes: Translational significance. Biochem Pharmacol. (2018) 153:123–33. doi: 10.1016/j.bcp.2018.01.024 29337002

[113] Xie C Lin Y Qi C Wang W Yuan Y Song D . Neuro-endocrine-immune regulation of metabolic homeostasis. Cytokine Growth Factor Rev. (2025) 85:165–78. doi: 10.1016/j.cytogfr.2025.08.001 40816942

[114] Prida E Álvarez-Delgado S Pérez-Lois R Soto-Tielas M Estany-Gestal A Fernø J . Liver brain interactions: Focus on Fgf21 a systematic review. Int J Mol Sci. (2022) 23:13318. doi: 10.3390/ijms232113318 36362103 PMC9658462

[115] Meng J Chen X Zhu Y . Whole-brain mapping reveals diet-dependent neuronal activation and selective resistance to exogenous Fgf21. Endocrinology. (2025) 167:bqaf176. doi: 10.1210/endocr/bqaf176 41287584 PMC12686946

[116] Mutsnaini L Kim CS Kim J Joe Y Chung HT Choi HS . Fibroblast growth factor 21 deficiency aggravates obesity-induced hypothalamic inflammation and impairs thermogenic response. Inflammation Res. (2019) 68:351–8. doi: 10.1007/s00011-019-01222-2 30863887

[117] Giralt M Gavaldà-Navarro A Villarroya F . Fibroblast growth factor-21, energy balance and obesity. Mol Cell Endocrinol. (2015) 418:66–73. doi: 10.1016/j.mce.2015.09.018 26415590

[118] Cheng Z Wei R Cao N Li Z Li M Liu M . Identification of hepatosensitive region and their neural connections in the hippocampus of rats. Folia Morphol (Warsz). (2022) 81:261–70. doi: 10.5603/FM.a2021.0020 33634834

[119] Woodie LN Melink LC Midha M de Araújo AM Geisler CE Alberto AJ . Hepatic vagal afferents convey clock-dependent signals to regulate circadian food intake. Science. (2024) 386:673–7. doi: 10.1126/science.adn2786 39509517 PMC11629121

[120] Bai W Zhu Z . Multiorgan crosstalk in Masld/Mash: From hepatic pathogenesis to systemic complications. Front Endocrinol (Lausanne). (2025) 16:1720780. doi: 10.3389/fendo.2025.1720780 41488134 PMC12756970

[121] Meroni M Longo M Paolini E Dongiovanni P . A narrative review about cognitive impairment in metabolic dysfunction-associated steatotic liver disease (Masld): Another matter to face through a holistic approach. J Adv Res. (2025) 68:231–40. doi: 10.1016/j.jare.2024.02.007 38369241 PMC11785580

[122] Capone F Vacca A Bidault G Sarver D Kaminska D Strocchi S . Decoding the liver-heart axis in cardiometabolic diseases. Circ Res. (2025) 136:1335–62. doi: 10.1161/circresaha.125.325492 40403112 PMC7617754

[123] Dimitriadis K Iliakis P Vakka A Pyrpyris N Pitsillidi A Tsioufis P . Effects of sympathetic denervation in metabolism regulation: A novel approach for the treatment of Masld? Cardiol Rev. (2025). doi: 10.1097/crd.0000000000000850 39750025

[124] Tilg H Moschen AR . Evolution of inflammation in nonalcoholic fatty liver disease: The multiple parallel hits hypothesis. Hepatology. (2010) 52:1836–46. doi: 10.1002/hep.24001 21038418

[125] Vachliotis ID Polyzos SA . The intriguing roles of cytokines in metabolic dysfunction-associated steatotic liver disease: A narrative review. Curr Obes Rep. (2025) 14:65. doi: 10.1007/s13679-025-00657-5 40794228 PMC12343754

[126] Liu M Chen R Zheng Z Xu S Hou C Ding Y . Mechanisms of inflammatory microenvironment formation in cardiometabolic diseases: Molecular and cellular perspectives. Front Cardiovasc Med. (2024) 11:1529903. doi: 10.3389/fcvm.2024.1529903 39877020 PMC11772298

[127] Stathori G Vlahos NF Charmandari E Valsamakis G . Obesity- and high-fat-diet-induced neuroinflammation: Implications for autonomic nervous system dysfunction and endothelial disorders. Int J Mol Sci. (2025) 26:4047. doi: 10.3390/ijms26094047 40362287 PMC12071462

[128] Solleiro-Villavicencio H Viurcos-Sanabria R Aguayo-Guerrero JA Pineda-Pérez PF Méndez-García LA . Inflammation: A key mechanism connecting metabolic-associated steatotic liver disease and systemic arterial hypertension. Front Immunol. (2025) 16:1620585. doi: 10.3389/fimmu.2025.1620585 40977717 PMC12446046

[129] Asimakidou E Saipuljumri EN Lo CH Zeng J . Role of metabolic dysfunction and inflammation along the liver-brain axis in animal models with obesity-induced neurodegeneration. Neural Regener Res. (2025) 20:1069–76. doi: 10.4103/nrr.Nrr-d-23-01770 38989938 PMC11438328

[130] Riazi K Galic MA Kentner AC Reid AY Sharkey KA Pittman QJ . Microglia-dependent alteration of glutamatergic synaptic transmission and plasticity in the hippocampus during peripheral inflammation. J Neurosci. (2015) 35:4942–52. doi: 10.1523/jneurosci.4485-14.2015 25810524 PMC6705378

[131] Fulton S Décarie-Spain L Fioramonti X Guiard B Nakajima S . The menace of obesity to depression and anxiety prevalence. Trends Endocrinol Metab. (2022) 33:18–35. doi: 10.1016/j.tem.2021.10.005 34750064

[132] Steinberg GR Carpentier AC Wang D . Mash: The nexus of metabolism, inflammation, and fibrosis. J Clin Invest. (2025) 135:e186420. doi: 10.1172/jci186420 40955659 PMC12435842

[133] Chai SY Zhang RY Fernandes G Zheng YM Wei L . Epidemiology of metabolic dysfunction-associated steatotic liver disease/metabolic dysfunction-associated steatohepatitis and associated cardiometabolic factors in adults in China (2013-2023): A systematic review and meta-analysis. World J Gastroenterol. (2025) 31:113608. doi: 10.3748/wjg.v31.i46.113608 41479652 PMC12754144

[134] Tilg H Petta S Stefan N Targher G . Metabolic dysfunction-associated steatotic liver disease in adults: A review. Jama. (2026) 335:163–74. doi: 10.1001/jama.2025.19615 41212550

[135] Rose JP Morgan DA Sullivan AI Fu X Inigo-Vollmer M Burgess SC . Fgf21 reverses Mash through coordinated actions on the CNS and liver. Cell Metab. (2025) 37:1515–29.e6. doi: 10.1016/j.cmet.2025.04.014 40367940 PMC12409791

[136] Cao Y Zhao Y Deng T Zhou Q Hu G Hu ZL . Hepatic acetyl-Coa metabolism modulates neuroinflammation and depression susceptibility via acetate. Cell Metab. (2025) 37:2185–201.e8. doi: 10.1016/j.cmet.2025.08.010 40992374

[137] Han Y Xu H Yao X Li Z Zhu C Li X . Molecular mechanisms and therapeutic progress in atherosclerosis: Bridging immune inflammation and precision medicine. Front Immunol. (2025) 16:1737662. doi: 10.3389/fimmu.2025.1737662 41562048 PMC12812629

[138] Girón GO Otero S Nikolaou PE Alcover S Ramos-Regalado L Zaragoza C . Atherogenic dyslipidaemia and residual cardiovascular risk: Understanding the link to heart disease. Eur J Clin Invest. (2026) 56:e70167. doi: 10.1111/eci.70167 41482986

[139] Ko S Anzai A Liu X Kinouchi K Yamanoi K Torimitsu T . Social bonds retain oxytocin-mediated brain-liver axis to retard atherosclerosis. Circ Res. (2025) 136:78–90. doi: 10.1161/circresaha.124.324638 39601150

[140] Zhou X Ma Z Cheng Q Jiang N Li J Zhan T . Sour neuronal signalling attenuates macrophage-mediated liver injury. J Hepatol. (2025) 83:738–49. doi: 10.1016/j.jhep.2025.02.026 40058705

[141] Felipo V . Hepatic encephalopathy: Effects of liver failure on brain function. Nat Rev Neurosci. (2013) 14:851–8. doi: 10.1038/nrn3587 24149188

[142] Shitara Y Maeda K Ikejiri K Yoshida K Horie T Sugiyama Y . Clinical significance of organic anion transporting polypeptides (Oatps) in drug disposition: Their roles in hepatic clearance and intestinal absorption. Biopharm Drug Dispos. (2013) 34:45–78. doi: 10.1002/bdd.1823 23115084

[143] Bookout AL de Groot MH Owen BM Lee S Gautron L Lawrence HL . Fgf21 regulates metabolism and circadian behavior by acting on the nervous system. Nat Med. (2013) 19:1147–52. doi: 10.1038/nm.3249 23933984 PMC3769420

[144] Teratani T Mikami Y Nakamoto N Suzuki T Harada Y Okabayashi K . The liver-brain-gut neural arc maintains the T(Reg) cell niche in the gut. Nature. (2020) 585:591–6. doi: 10.1038/s41586-020-2425-3 32526765

[145] Nguyen TL Vieira-Silva S Liston A Raes J . How informative is the mouse for human gut microbiota research? Dis Model Mech. (2015) 8:1–16. doi: 10.1242/dmm.017400 25561744 PMC4283646

[146] Takahashi Y Soejima Y Fukusato T . Animal models of nonalcoholic fatty liver disease/nonalcoholic steatohepatitis. World J Gastroenterol. (2012) 18:2300–8. doi: 10.3748/wjg.v18.i19.2300 22654421 PMC3353364

[147] Mardinoglu A Boren J Smith U Uhlen M Nielsen J . Systems biology in hepatology: Approaches and applications. Nat Rev Gastroenterol Hepatol. (2018) 15:365–77. doi: 10.1038/s41575-018-0007-8 29686404

[148] Moretti R Caruso P Gazzin S . Non-alcoholic fatty liver disease and neurological defects. Ann Hepatol. (2019) 18:563–70. doi: 10.1016/j.aohep.2019.04.007 31080056

[149] Lonardo A Nascimbeni F Ballestri S Fairweather D Win S Than TA . Sex differences in nonalcoholic fatty liver disease: State of the art and identification of research gaps. Hepatology. (2019) 70:1457–69. doi: 10.1002/hep.30626 30924946 PMC6766425

[150] Trapecar M Wogram E Svoboda D Communal C Omer A Lungjangwa T . Human physiomimetic model integrating microphysiological systems of the gut, liver, and brain for studies of neurodegenerative diseases. Sci Adv. (2021) 7:eabd1707. doi: 10.1126/sciadv.abd1707 33514545 PMC7846169

[151] Weinstein G Zelber-Sagi S Preis SR Beiser AS DeCarli C Speliotes EK . Association of nonalcoholic fatty liver disease with lower brain volume in healthy middle-aged adults in the Framingham study. JAMA Neurol. (2018) 75:97–104. doi: 10.1001/jamaneurol.2017.3229 29159396 PMC5833484

[152] Romero-Gómez M Zelber-Sagi S Trenell M . Treatment of nafld with diet, physical activity and exercise. J Hepatol. (2017) 67:829–46. doi: 10.1016/j.jhep.2017.05.016 28545937

[153] Bonaz B Bazin T Pellissier S . The vagus nerve at the interface of the microbiota-gut-brain axis. Front Neurosci. (2018) 12:49. doi: 10.3389/fnins.2018.00049 29467611 PMC5808284

[154] Tillman EJ Rolph T . Fgf21: an emerging therapeutic target for non-alcoholic steatohepatitis and related metabolic diseases. Front Endocrinol (Lausanne). (2020) 11:601290. doi: 10.3389/fendo.2020.601290 33381084 PMC7767990

